# Profiling of amines in biological samples using polythioester-functionalized magnetic nanoprobe

**DOI:** 10.3389/fbioe.2022.1103995

**Published:** 2023-01-04

**Authors:** Yuming Qiu, Mo Zhang, Zhizhen Lai, Renjun Zhang, Hongtao Tian, Shuai Liu, Dan Li, Jiang Zhou, Zhili Li

**Affiliations:** ^1^ Institute of Basic Medical Sciences, Chinese Academy of Medical Sciences and Peking Union Medical College, Beijing, China; ^2^ Analytical Instrumentation Center, College of Chemistry and Molecular Engineering, Peking University, Beijing, China

**Keywords:** magnetic particle, polythioester, chemoselective capture, amine, biomarker

## Abstract

**Introduction:** The metabolic balance of amines is closely related to human health. It remains a great challenge to analyze amines with high-throughput and high-coverage.

**Methods:** Polythioester-functionalized magnetic nanoprobes (PMPs) have been prepared under mild conditions and applied in chemoselective capture of amides. With the introduction of polythioester, PMPs demonstrate remarkably increased capture efficiency, leading to the dramatically improved sensitivity of mass spectrometry detection.

**Results:** The analysis method with PMPs treatment has been applied in rapid detection of more than 100 amines in lung adenocarcinoma cell lines, mouse organ tissues, and 103 human serum samples with high-throughput and high-coverage. Statistical analysis shows that arginine biosynthesis differed between lung adenocarcinoma cell lines.

**Discussion:** Phenylalanine, tyrosine and tryptophan biosynthesis differed between tissues. The combination indicators demonstrate a great diagnostic accuracy for distinguishing between health and lung disease subjects as well as differentiating the patients with benign lung disease and lung cancer. With powerful capture ability, low-cost preparation, and convenient separation, the PMPs demonstrate promising application in the intensive study of metabolic pathways and early diagnosis of disease.high-throughput and high-coverage. Here, polythioester-functionalized magnetic nanoprobes (PMPs) have been prepared under mild conditions and applied in chemoselective capture of amides. With the introduction of polythioester, PMPs demonstrate remarkably increased capture efficiency, leading to the dramatically improved sensitivity of mass spectrometry detection. The analysis method with PMPs treatment has been applied in rapid detection of more than 100 amines in lung adenocarcinoma cell lines, mouse organ tissues, and 103 human serum samples with high-throughput and high-coverage. Statistical analysis shows that arginine biosynthesis differed between lung adenocarcinoma cell lines. Phenylalanine, tyrosine and tryptophan biosynthesis differed between tissues. The combination indicators demonstrate a great diagnostic accuracy for distinguishing between health and lung disease subjects as well as differentiating the patients with benign lung disease and lung cancer. With powerful capture ability, low-cost preparation, and convenient separation, the PMPs demonstrate promising application in the intensive study of metabolic pathways and early diagnosis of disease.

## 1 Introduction

Amines play an important role in a range of metabolic processes in living organisms (Vissers et al., 2005; Pegg, 2009; Huang et al., 2011; Bröer and Bröer, 2017; Greene et al., 2019; Sayé et al., 2019), such as transcription and translation of genes, structural regulation of chromatin and proteins, cell growth and migration, as well as in the synthesis and metabolism of nucleic acids (Tremblay, 2007; Wallace, 2009; Doeun et al., 2017; Zhang et al., 2021). Dysregulation of these metabolites may lead to various diseases, indicating the potential application of amines in the prediction of disease processes, and prognosis assessment (Soga et al., 2011; Zembron-Lacny et al., 2021; Wu et al., 2022). Therefore, the analysis of amines in biological samples provides important value for metabonomics and disease diagnosis.

Currently, due to the complex matrix of biological samples and the wide dynamic range of amines, it remains a great challenge to analyze amines in biological samples with high-throughput and high-coverage. The most common analytical methods for amines combine derivatization, chromatographic separation techniques, and mass spectrometry (MS) detection (Moore and Stein, 1948; Shimbo et al., 2009; Ayon et al., 2019). The stable isotope-coded derivatization method using 1-bromobutane and 1-bromobutane-4,4,4-d3 was applied for comparative analysis of amino acids in human serum (Sakaguchi et al., 2015). The needle-tip device coupled ultrafast in-fiber extraction and derivatization strategy to gas chromatography-mass spectrometry to simultaneously quantify twenty amines in urine (Yu et al., 2022). Although the sensitivity of detection could be remarkably improved *via* derivatization and separation, these analytical methods suffer from the excessive derivatization reagents and by-products. The universal probe to realize the capture, derivatization, and separation of amines simultaneously is urgent for the profiling of amines with high-throughput and high-coverage.

Combining large specific surface area, good biocompatibility, flexible functionalization, and convenient operation, magnetic nanomaterials are widely used in bioseparation (Son et al., 2005; Carlson and Cravatt, 2007; Li et al., 2013; Khizar et al., 2021; Gessner et al., 2022). Magnetic separation processes are generally mild and non-destructive for biological analytes such as proteins, peptides, and protein complexes which are prone to disintegrate during traditional column chromatographic separations (Shao et al., 2012). The versatile probe of magnetic particles provides chemoselective capture and derivatization of target compounds in the matrix background, and then improves ionization of the captured and tagged compounds after release from the probes (Garg et al., 2018; Lin et al., 2020). With chemoselective capture and efficient separation of target analytes, the magnetic separation process combined with other analytical methods is suitable for high-throughput operation and analysis with high-coverage (Lin et al., 2021; Liu et al., 2022).

**FIGURE 1:**
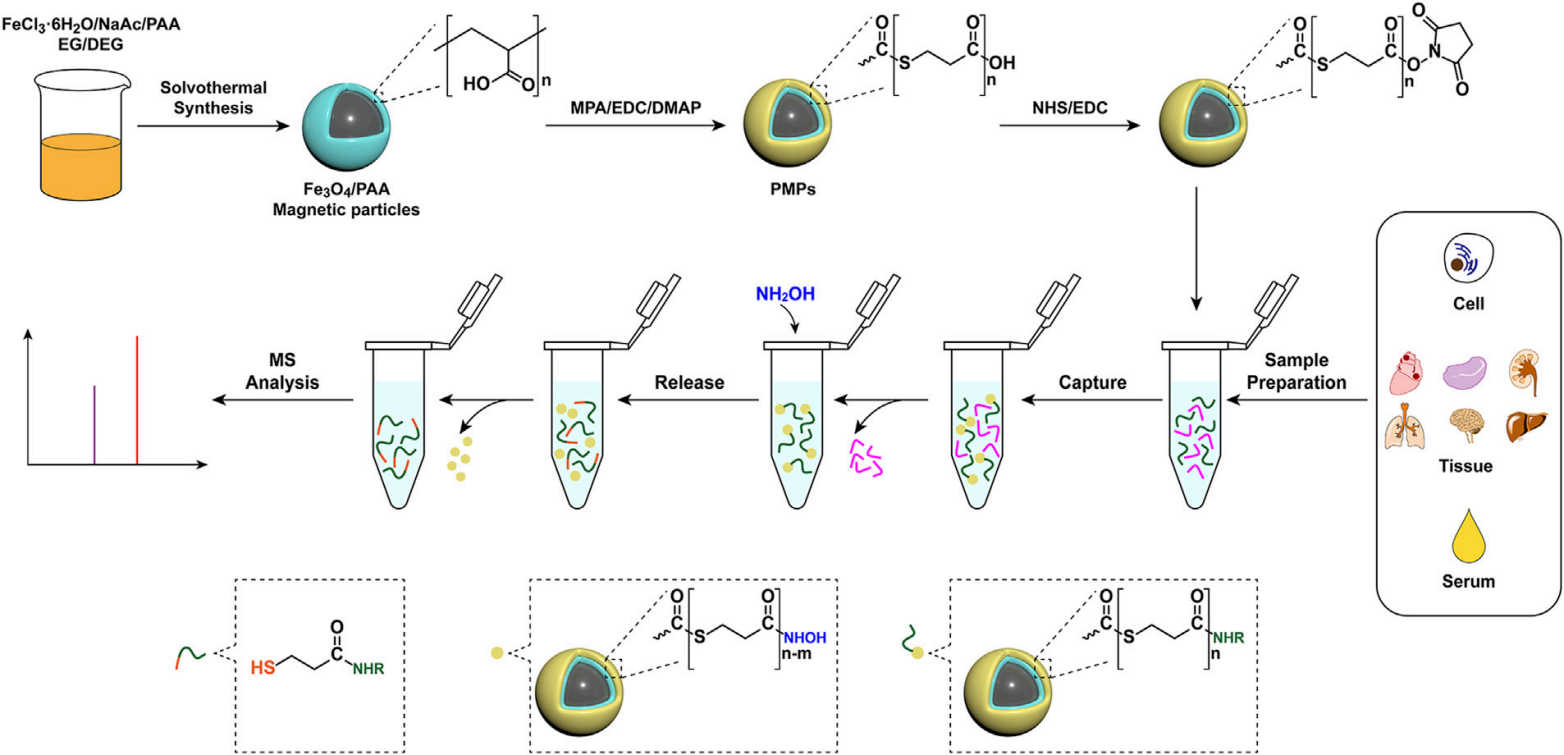
Schematic illustration of the preparation of PMPs and their application in chemoselective capture, derivatization, release, and detection of amino metabolites from biological samples.

Polymers-assisted magnetic nanoparticles have playing great roles in biomedical applications due to the manipulation of physical, chemical and biological properties of magnetic nanoparticles *via* introduction of polymers (Li et al., 2020). In this study, polythioester-functionalized magnetic nanoprobes (PMPs) were designed for chemoselective capture and derivatization of amines in complex biological samples by the specific reaction between activated carboxyl groups and amino groups (Figure 1). The amine derivatives could be released in pure solvent *via* cleavage of thioester bond by hydroxylamine, leading to the efficient separation of target metabolite from the complex matrix. The PMPs treatment improved the selectivity of the amine and reduced the interference of complex matrices. With remarkably improved sensitivity as well as excellent stability and accuracy, the analysis method with PMPs treatment has been applied in the detection of amines in lung adenocarcinoma cells, healthy mouse organ tissues, and human serum, demonstrating the feasibility of PMPs for capture and detection of amines in complex biological samples with high-throughput and high-coverage.

## 2 Materials and methods

### 2.1 Materials and chemicals

All standard amines, hydroxylamine (NH_2_OH, 50 wt. % in H_2_O), 3-mercaptopropionic acid (MPA), 4-dimethylaminopyridine (DMAP), and N-hydroxysuccinimide (NHS) were purchased from Sigma-Aldrich. L-glutamic acid-^13^C5 (L-Glu-^13^C5), L-valine-^13^C5,^15^N (L-Val-^13^C5,^15^N), L-tyrosine-^13^C6 (L-Tyr-^13^C6), L-arginine-^13^C6 hydrochloride (L-Arg-^13^C6), L-glycine-^13^C2 (L-Gly-^13^C2) were purchased from Toronto Research Chemical. Methanol (MeOH, HPLC, MS), Acetonitrile (ACN, HPLC, MS), N,N-Dimethylformamide (DMF, HPLC), and Dichloromethane (DCM, HPLC) were purchased from Thermo Fischer. Polyacrylic acid (PAA), Ethylene glycol (EG), and Diethylene glycol (DEG) were purchased from Shanghai Aladdin Biochemical Technology Co., Ltd. 1-(3-Dimethylaminopropyl)-3-ethylcarbodiimide hydrochloride (EDC), and sodium acetate (NaAc) were purchased from Shanghai Macklin Biochemical Co., Ltd. FeCl_3_.6H_2_O was purchased from Sinopharm Chemical Reagent Co., Ltd.

### 2.2 Synthesis of Fe_3_O_4_/PAA magnetic particles

FeCl_3_.6H_2_O (1.08 g), NaAc (4.00 g), and PAA (100 mg) were dissolved in a mixture of EG (14.0 ml) and DEG (24.0 ml) and stirred magnetically at room temperature for 1 h. Then the mixture was poured into the autoclave and heated at 200°C for 15 h to obtain Fe_3_O_4_/PAA magnetic beads. After the temperature of the autoclave was cooled to room temperature, Fe_3_O_4_/PAA particles were obtained by magnetic separation and rinsed alternately with water and ethanol. The cleaned Fe_3_O_4_/PAA beads were dried at 50°C.

### 2.3 Synthesis of PMPs

1 mg Fe_3_O_4_/PAA magnetic particles were dispersed in acetonitrile (300 μL) and reacted with EDC (12.78 mg) and DMAP (1.22 mg) by shaking at room temperature for 6 h to obtain PMPs.

### 2.4 Activation of PMPs

The PMPs were dispersed in 300 μL DCM and reacted with EDC (12.78 mg) and NHS (7.67 mg) by shaking for 12 h (Supplementary Figure S1A) at room temperature.

### 2.5 The capture and release of amines

The treated biological samples were reacted with activated PMPs (3 mg) in a mixture of 1 ml DMF/MeOH (v/v, 9/1) and 1.5 μL TEA for 30 min at room temperature. After the reaction finished, the supernatant was discarded. The beads were washed six times with H_2_O and DMF alternately, followed by the addition of 500 μL MeOH/H_2_O/ACN (v/v/v, 1/1/3) and 6 μL NH_2_OH (50 wt. % in H_2_O) and shaking for 25 min. Then the PMPs were magnetic separated and the raffinate was centrifuge (4°C, 15,000 × g) for 15 min. 100 μL of the raffinate was added into 400 μL acetonitrile for dilution and MS detection.

### 2.6 Method validation

Method validation is performed to demonstrate the reliability of various parameters and the developed method. The linearity, precision, accuracy, the limit of quantification, and the limit of detection were tested.

### 2.7 Linearity

A mixed stock solution of amine standards was prepared (Supplementary Table S1). The gradient dilution of the mixed amines standard solution was added to the same concentration of internal standard (IS) solution for quantification. The absolute intensity ratio of amines standards to IS was ordinate values, and the concentration ratio was abscissa values. The calibration curve was plotted, and the equation of calibration curve and coefficient of determination (*R* ^2^) were obtained.

### 2.8 Limit of quantification and limit of detection

The mixture of amine standards was prepared by gradient dilution. The concentration at a signal-to-noise ratio (S/N) of 10 was used as the limit of quantification (LOQ) and the concentration at a signal-to-noise ratio (S/N) of 3 was used as the limit of detection (LOD) by MS detection.

### 2.9 Recovery

Standard solutions with low, medium and high concentrations were mixed with ovalbumin and IS solution (Supplementary Table S2). The ovalbumin (50 mg/ml) was served as a substitute for the serum system. The concentration of the amine was obtained by calibration curve, and the ratio of the calculated concentration to the actual concentration was the recovery.

### 2.10 Precision

The precision of the method was assessed by analyzing the relative standard deviation (RSD) of intra-day and inter-day concentrations of amines in serum samples.

### 2.11 Data analysis

All mass spectra were acquired using ftmsControl 3.0.0 (Bruker Daltonics, Billerica, MA, United States) software. Molecular information (mass-to-charge ratio and absolute intensity) of amines in the mass spectra were set up and extracted using Data Analysis 4.0 software. Substance identification was performed by precise molecular weights from high-resolution mass spectrometry and library search matching identification through an open-source database (http://www.hmdb.ca/). GraphPad Prism 8 was used for scatter plotting and the Kruskal-Wallis test. Binary logistic regression analysis was performed by IBM SPSS Statistics 26 to screen potential biomarkers, and receiver operating characteristic (ROC) was used to evaluate the diagnostic ability of potential biomarkers.

## 3 Results

### 3.1 Characterization of PMPs

**FIGURE 2:**
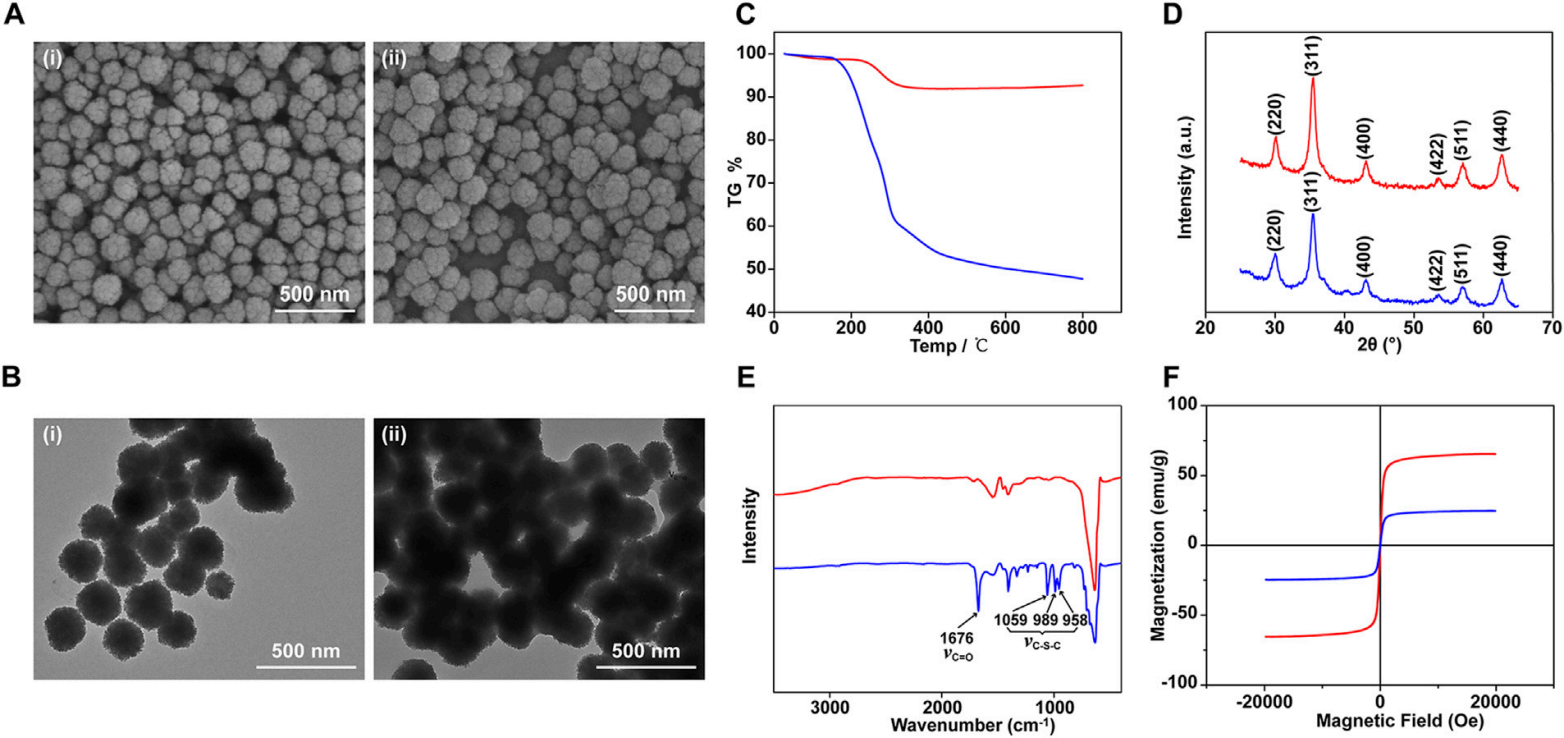
Characterization of Fe_3_O_4_/PAA and PMPs. SEM **(A)** and TEM **(B)** images of Fe_3_O_4_/PAA magnetic beads (i) and PMPs (ii); TG curves **(C)**, X-rays diffractogram **(D)**, IR curves **(E)**, and magnetic hysteresis curves **(F)** of Fe_3_O_4_/PAA (red curves) and PMPs (blue curves).

The preparation procedure for PMPs is schematically illustrated in Figure 1. Briefly, Fe_3_O_4_/PAA particles were prepared by a modified solvothermal reaction. Then the Fe_3_O_4_/PAA particles were coated by polythioesters *via* polymerization of 3-mercaptopropionic acid to form core-shell magnetic particles (PMPs). The typical scanning electron microscope (SEM) images and transmission electron microscopy (TEM) of magnetic particles are shown in Figures 2A, B. The Fe_3_O_4_/PAA particles displayed uniform size with a mean diameter between 200 nm and 300 nm. After coated with polythioester, the size was increased and the shell thickness was about 10 nm. As shown in the thermogravimetric analysis of Fe_3_O_4_/PAA particles and PMPs (Figure 2C), the weight loss of Fe_3_O_4_/PAA particles was 8% with a step around 220°C, indicating the corresponding decomposition temperature of PAA. With the introduction of polythioester, the weight loss was increased to 47% with a pronounced step around 200°C. Figure 2D shows the X-ray diffraction patterns of magnetic particles. The similar profiling of diffraction patterns of Fe_3_O_4_/PAA particles and PMPs were observed with a series of characteristic peaks in good accordance with the inverse cubic spinel phase of Fe_3_O_4_ (magnetite, JCPDS card no. 85-1436), including (220), (311), (400), (422), (511), and (440). The results indicated that the crystal lattice structure of Fe_3_O_4_ was not changed by polymer functionalization. The infrared spectra of magnetic particles are shown in Figure 2E. The characteristic absorption peak at 1676 cm^−1^ corresponds to the stretching vibration of C=O. After coated with polythioester, several new absorption peaks were added between 958 cm^−1^ and 1059 cm^−1^. These peaks correspond to the stretching vibration of C-S-C, indicating the thioester bond formation. As shown in Figure 2F, the superparamagnetic responsiveness of Fe_3_O_4_/PAA and PMPs allowed them to be easily separated from the solution. With polythioester modifications, the saturation magnetization value of PMPs was decreased from 65.39 to 24.68 emu/g. The structure of polythioester was identified by mass spectrometry (Supplementary Figure S1 and Supplementary Table S1), the major ions serie with a mass difference of 88 Da between peaks indicated that the repeating unit of the polythioester was -S-CH_2_-CH_2_-CO-.

### 3.2 Applications in capture and analysis of standards

The chemoselective capture of standards by PMPs was based on the condensation reaction between amino and NHS-activated carboxyl groups. The standard derivatives were released through the cleavage of the thioester bond by hydroxylamine (Figure 1) and detected by LC-MS/MS. The reaction conditions of polymerization, chemoselective capture, and release were optimized by comparing the peak area of derivatives of standards.

**FIGURE 3:**
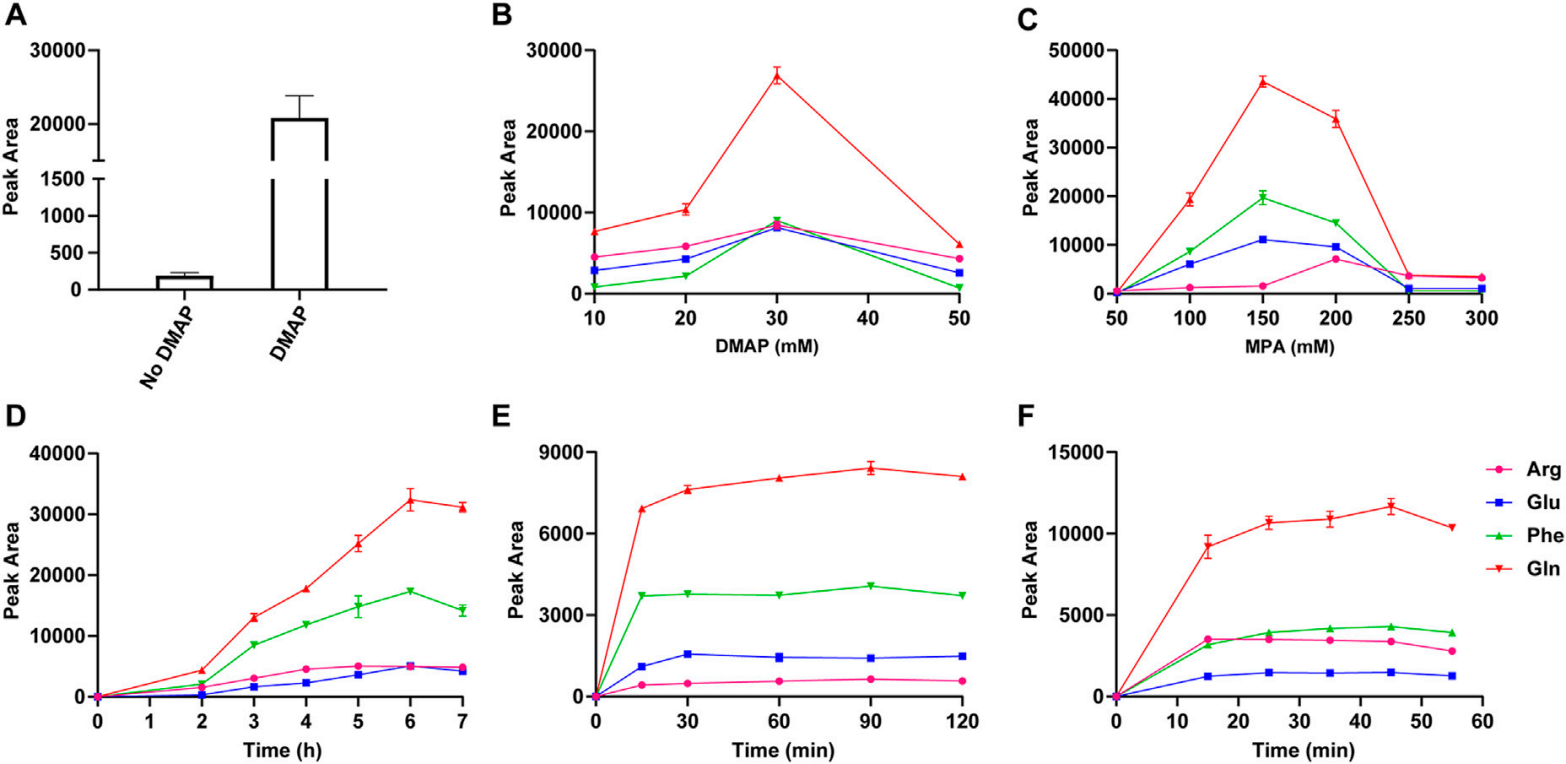
The peak areas of valine derivatives detected using LC-MS, which were released from magnetic particles prepared with or without DMAP **(A)**; The optimization of the concentration of DMAP **(B)** and MPA **(C)**, the reaction time of polymerization **(D)**, capture **(E)**, and release **(F)** by comparing the peak areas of standard derivatives released from PMPs.

The addition of DMAP is crucial for the polymerization reaction and the capture efficiency of PMPs. With EDC as a condensation agent, the condensation reaction of MPA was carried out in the dichloromethane solution. The oligomers and by-products were generated in the transparent solution (Supplementary Figures S2A–F). With the addition of DMAP, polythioester precipitated in 6 h, indicating that self-polymerization of MPA was developed with the catalysis of DMAP (Supplementary Figures S2B, C, G–I). As shown in Figure 3A, the peak area of derivatives cleaved from PMPs was increased by 2 orders of magnitude compared with that of magnetic particles prepared without DMAP, indicating the dramatically enhanced capture efficiency of PMPs with the introduction of polythioester. Figures 3B–D shows that the peak area of standard derivatives increases first and then declines with increasing concentration of DMAP and MPA as well as the prolonged reaction time, which could lead to excessive polymerization and might be adverse for detection due to the interference of byproducts. Therefore, the concentrations of DMAP and MPA were optimized as 30 mM and 200 mM respectively and 6 h is the optimal reaction time for polymerization.

The conditions of chemoselective capture and release of standards were investigated (Figures 3E, F and Supplementary Figures S3A–E). The peak areas of standard derivatives increased with a prolonged reaction time of capture from 0 to 30 min and stayed broadly flat with proceeding on increased reaction time. Thus, 30 min was an appropriate duration for capture. The reaction time of carboxyl group activation was optimized as 10 h. Served as a proton receptor for condensation reaction between PMPs and standards, TEA was added and the concentration was optimized as 1.5 μL/ml in DMF/MeOH (v/v, 9/1). The excessive TEA might accelerate the deactivation process, which was adverse for capture. The standard derivatives were released from PMPs via cleavage of thioester bond by hydroxylamine. The peak areas of standard derivatives increased with the mounting concentration of hydroxylamine from 2 μL/ml to 12 μL/ml and stayed broadly flat with proceeding on increased concentration. Thus the concentration of hydroxylamine was optimized as 12 μL/ml in MeOH/H_2_O/ACN (v/v/v, 1/1/3). There was no consistent trend for detection of all the standards with an increased reaction time of release from 0 to 55 min. Factoring in the results, the release time was optimized as 15 min.

The quantitative analysis of standards was based on direct infusion Electron Spray Ionization-Fourier Transform Ion Cyclotron Resonance Mass Spectrometer (ESI-FTICR MS). The theoretical m/z values, observed m/z values, and mass errors of the standard derivatives in negative ion mode are shown in Supplementary Table S2. The mass error was less than .0001 Da and the relative mass deviation was less than 1 ppm. The analysis method was of excellent linearity with the correlation coefficients higher than .99. The limits of quantification were between .0095 and .6075 μM and the limits of detection were between .0032 and .6075 μM. The intra-day relative standard deviations (RSD) of the method ranged from .34% to 4.42% and the inter-day RSDs ranged from 1.51% to 14.88%, indicating the excellent stability of the analysis method. The recoveries of the standards at the three levels ranged from 76.17% to 115.32%, suggesting that the method was accurate and reliable.

### 3.3 Applications in capture and analysis of amines in biological samples

**FIGURE 4:**
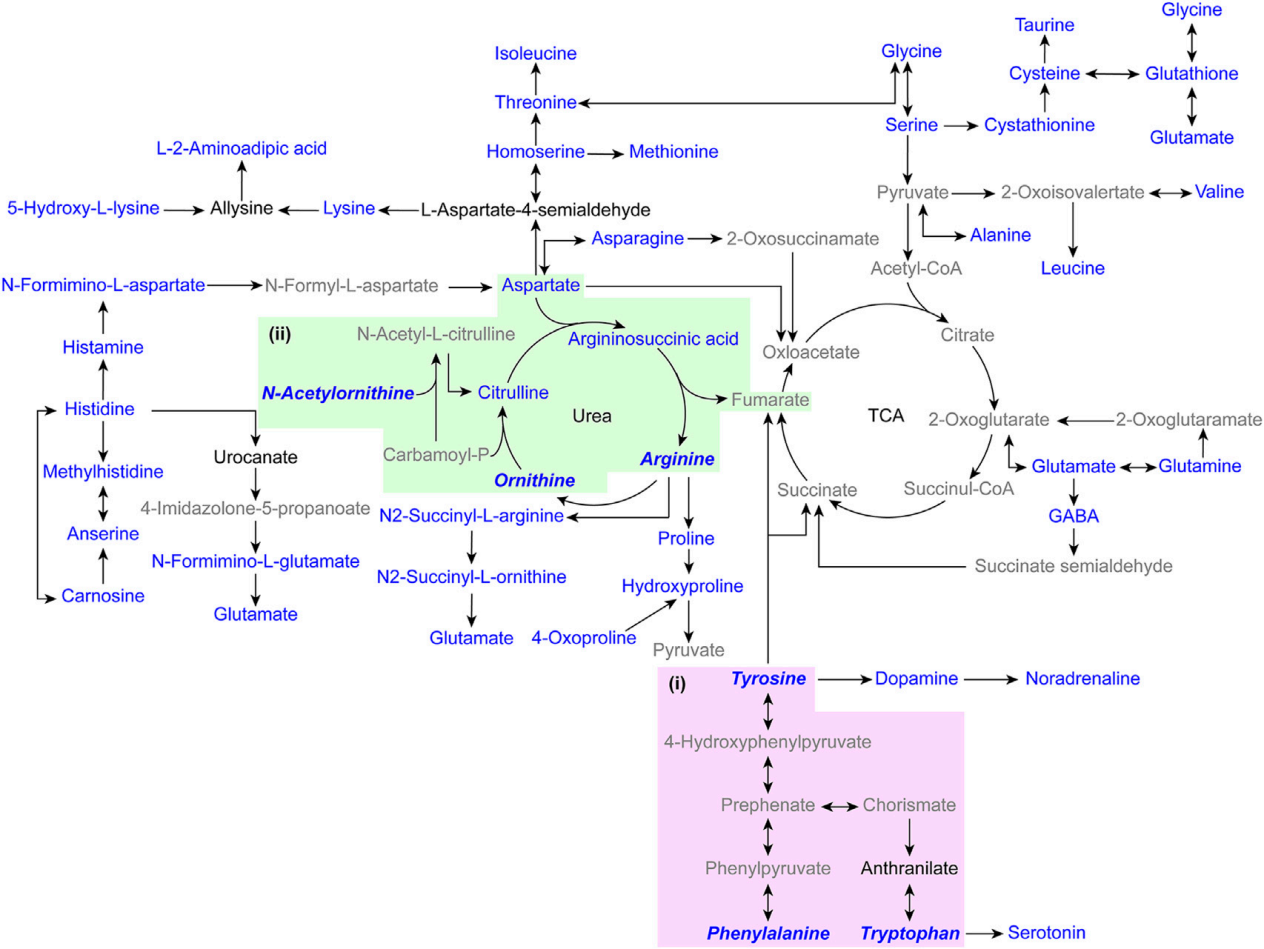
Pathway map of the detected metabolites in biological samples. **(i)** Phenylalanine, tyrosine and tryptophan biosynthesis. **(ii)** Arginine biosynthesis. (Blue indicates captured amino compounds, blue italic bold indicates compounds with differences in the pathway, black indicates untrapped amino compounds, and gray indicates compounds without amino groups).

With excellent stability and accuracy, the method was further applied in the analysis of amines in biological samples. Intracellular amines from three lung adenocarcinoma cell lines, HCC827, NCI-H1650, and NCI-H2228 were analyzed. 26 amino acids and cystathionine (dipeptide) were quantified (Supplementary Table S3). As shown in Figure 4, the intracellular amines were involved in complicated and inconstant metabolic pathways, including several amino acid biosynthesis pathways, amino acid metabolism pathways, and the urea cycle. Kruskal-Wallis test for amines revealed that ornithine, N-acetylornithine, and arginine in the arginine biosynthesis pathway differed between lung adenocarcinoma cells (Figure 4ii, Supplementary Figure S4, and Supplementary Table S4), which was similar to previous reports (Lim et al., 2018).

The PMPs were applied in the analysis of amino metabolites from mouse tissues, including mouse heart tissue (*n* = 5), liver tissue (*n* = 5), spleen tissue (*n* = 5), lung tissue (*n* = 5), kidney tissue (*n* = 5), and brain tissue (*n* = 5). 25 amino acids, cystathionine (dipeptide), and alanylglutamine (dipeptide) were quantified (Supplementary Table S5). The amines are involved in several pathways, such as the TCA cycle, urea cycle, amino acid biosynthesis pathway, and amino acid metabolism pathway (Figure 4). The Kruskal-Wallis test showed that amines were significantly different between tissues (Supplementary Table S6). Among them, aromatic amino acids were less abundant in brain tissues than in other tissues (Supplementary Figure S5), which were involved in phenylalanine, tyrosine and tryptophan biosynthesis (Figure 4i). The results indicate that the analysis method demonstrates extensive application in the detailed study of the biochemical landscape.

**FIGURE 5:**
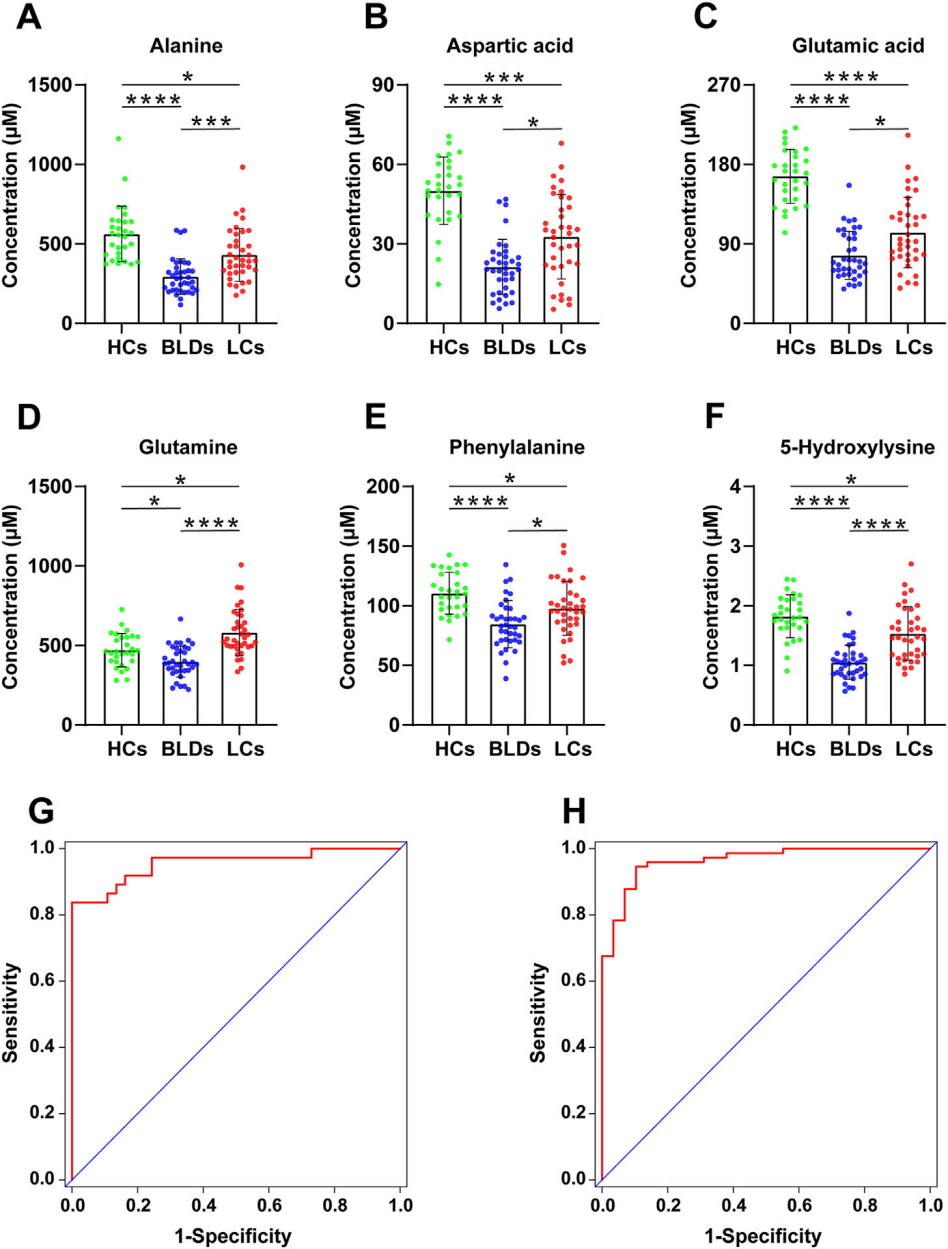
The scatter plots of Alanine **(A)**, Aspartic acid **(B)**, Glutamic acid **(C)**, Glutamine **(D)**, Phenylalanine **(E)**, and 5-Hydroxylysine **(F)** from HCs, BLDs, and LCs (*, *p* < .05, **, *p* < .01, ***, *p* < .001, ****, *p* < .0001). Receiver operating characteristic curves for HCs and patients **(G)** as well as BLDs and LCs **(H)**.

The method was further applied in the analysis of amines in serum and biomarker discovery. As shown in Supplementary Table S7, the samples from 29 healthy controls (HCs), 37 patients with benign lung diseases (BLDs), and 37 patients with lung cancer (LCs) were processed and analyzed, and a QC sample was added to every 12 samples to verify the stability of the experimental process. 30 amino acids as well as cystathionine (dipeptide) and alanylglutamine (dipeptide) were quantified (Supplementary Table S8). Statistical analysis in this study showed that the profile of serum amines was significantly altered in patients with benign lung disease and lung cancer patients. Healthy controls, patients with benign lung disease, and patients with lung cancer were grouped according to sex and age (<49 and ≥49), respectively. The subjects were matched for age and sex by Wilcoxon rank-sum tests (Supplementary Figure S6). Kruskal-Wallis test was performed for each amine (Supplementary Table S9). Alanine, aspartic acid, glutamine, glutamate, phenylalanine, and 5-hydroxylysine were significantly different among the three groups (Figures 5A–F).

With PMPs treatment, a total of 103 amines were detected from biological samples (Supplementary Table S10). The amino acids were used to differentiate between healthy controls, patients with benign lung disease, and patients with lung cancer. Screening of biomarkers by the binary logistic regression model, the diagnostic ability of potential markers was evaluated by ROC analysis (Figures 5G, H). Panel 1 (alanine, glutamine, glutamic acid, 5-hydroxylysine) could distinguish well between healthy controls and lung diseases (including benign lung diseases and lung cancer) with an AUC of .964 (95%CI [.931-.997]), the sensitivity of 95.9% and the specificity of 82.8% (Table 1). Panel 2 (aspartic acid, glutamine, glutamic acid, phenylalanine, 5-hydroxylysine) distinguished BLDs from LCs with an AUC of .956 (95% CI [.910-1.000]), the sensitivity of 86.5%, and the specificity of 86.5% (Table 1). The results indicate that PMPs are promising nanoprobes for chemoselective capture of amines and biomarker discovery.

**TABLE 1:** The diagnostic efficiency of Panel 1 (combinated marker of alanine, glutamine, glutamic acid, and 5-hydroxylysine) and Panel 2 (combinated marker of aspartic acid, glutamine, glutamic acid, phenylalanine, and 5-hydroxylysine).

	AUC (95% CI)	Sensitivity (%)	Specificity (%)
HCs vs. BLDs + LCs	0.964 (.931–.997)	95.9	82.8
Panel 1 (alanine + glutamine + glutamic acid+5-hydroxylysine)			
BLDs vs. LCs	0.956 (.910–1.000)	86.5	86.5
Panel 2 (aspartic acid + glutamine + glutamic acid + phenylalanine+5-hydroxylysine)			

## 3 Discussion

Given the importance of amino compounds in life processes, a large number of studies have been devoted to the analysis of amino compounds in biological samples (Puchades-Carrasco et al., 2016; Vanhove et al., 2018). In this study, amino compounds in biological samples were captured by covalent bonding using PMPs and detected by the ESI-FTICR MS technique. The new analytical method established has good accuracy, precision, linearity, and stability for the study of amino compounds in complex matrices. Ultimately, amino compounds were analyzed in six mouse tissues, three lung adenocarcinoma cell lines, and 103 human sera. Compared with other methods for the analysis of amino compounds, this method does not require pre- or post-column derivatization and chromatographic separation, shortens the detection time by direct injection, enables high-throughput analysis of complex samples, and improves the selectivity of magnetic beads for amino compounds by covalent bonding.

Phenylalanine, tyrosine and tryptophan biosynthesis differ between tissues. Phenylalanine, tyrosine, and tryptophan are known as aromatic amino acids because of the phenyl ring in their chemical structure, and only in the liver can all three be completely degraded to CO_2_. Aromatic amino acids are substrates for the synthesis of important neurotransmitters, tryptophan is a precursor of serotonin and tyrosine is a precursor of dopamine and norepinephrine in the brain (Fernstrom, 2013). Tryptophan and tyrosine cannot be biosynthesized in any brain cell and can only be taken up from the blood through the blood-brain barrier (He and Wu, 2020). The central nervous system lacks phenylalanine hydroxylase to convert phenylalanine to tyrosine and can only take up phenylalanine from the circulation by the kidneys and liver, which act on phenylalanine hydroxylase to release tyrosine (Hufton et al., 1995). These may be related to the lower level of aromatic amino acids in brain tissue compared to other tissues. A stable metabolic state of aromatic amino acids is essential for the maintenance of normal physiological functions of the organism.

Abnormal metabolism of amino compounds has been reported to occur in patients with lung adenocarcinoma (Wen et al., 2013). In the present study, the arginine biosynthetic pathway differed between different lung adenocarcinoma cell lines. Arginine is produced by the urea cycle during the conversion of ammonia and aspartate to urea (Luengo et al., 2017). The rate-limiting enzyme in arginine biosynthesis is argininosuccinate synthase 1 (ASS1), which catalyzes the formation of argininosuccinate from aspartate and citrulline. Argininosuccinate lyase (ASL) then cleaves argininosuccinate to arginine and fumarate (Luengo et al., 2017). Since ASS1-deficient tumor cells rely on extracellular arginine for survival, arginine deprivation is a therapeutic strategy for these cancers (Tsai et al., 2012). For example, recombinant human arginase (rhARG) cleaves arginine to urea and ornithine, and treatment with rhARG reduces mTORC1 activity and induces cytotoxicity and apoptosis in non-small cell lung cancer cells (Shen et al., 2017). The interaction of amino compounds in the metabolic process affects the metabolic process, which can provide insight into the differences in amino acid metabolism and changes in metabolic pathways that exist between disease cell lines and normal cell lines and promote progress in the study of disease mechanisms.

Amino acids play an important role in tumor metabolism and they are essential for tumor growth and proliferation (Tsun and Possemato, 2015). Statistical analysis in this study showed that the profile of serum amino compounds was significantly altered in patients with benign lung disease and lung cancer patients. The concentration levels of glycine, alanine, aspartate, glutamine, glutamate, phenylalanine, and 5-hydroxylysine were significantly different between healthy controls and patients with lung cancer, between patients with benign lung disease and patients with lung cancer, and between healthy controls and patients with benign lung disease. Although tumor markers have been widely used to detect lung cancer and to assess clinical conditions, these markers do not always predict correctly due to insufficient specificity and sensitivity; therefore, the use of a combination of two or more independent tumor markers is necessary for clinical applications (Ando et al., 2003). The results of this experiment showed that different combinations of amino acids can be used to diagnose the pathophysiological state of the subject. Significantly decreased glutamate levels and significantly elevated glutamine levels suggest that metabolic changes during lung carcinogenesis may lead to a shift and increase in glutamine metabolism. High levels of glutamine in the blood provide a source of carbon and nitrogen to support biosynthesis, bioenergetics, and cellular homeostasis, which cancer cells use to drive tumor growth (Altman et al., 2016). Glutamine is transported into cells by the transporter protein SLC1A5 (solute carrier family 1 neutral amino acid transporter protein member 5) (Wise and Thompson, 2010) and can be used for biosynthesis or exported extracellularly *via* reverse transporter proteins. L-Type amino acid transporter protein 1 (LAT1, a heterodimer of SLC7A5 and SLC3A2) transports glutamine extracellularly to be exchanged with essential amino acids such as leucine exchange (Nicklin et al., 2009), which leads to the activation of the mTOR signaling pathway, thereby promoting protein synthesis and inhibiting autophagy (Choi and Park, 2018). The dependence of cancer cells on glutamine metabolism could be developed as a new therapeutic target. Glycine, alanine, aspartate, phenylalanine, and 5-hydroxylysine concentration levels were also significantly reduced, which may reflect the hypermetabolic state and the dramatic increase in amino acid requirements during tumor development. There are few reports on changes in serum levels of free 5-hydroxylysine, a hydroxylated derivative of lysine, which is produced in its free form by hydrolytic degradation of collagen. Collagen is a major component of the tumor microenvironment and is involved in tumor fibrosis, but how collagen is catabolized in tumors has not been elucidated (Xu et al., 2019). It is expected to clarify the biological mechanisms of collagen anabolism and catabolism in tumors, and thus the role of 5-hydroxylysine in tumor development.

In summary, polythioesters functionalized magnetic nanoparticles were synthesized for the chemoselective capture of amines. With powerful capture ability, low-cost preparation, and convenient magnetic separation, the PMPs were applied in the development of an analysis method to detect the amines in biological samples. The results revealed differences in levels of the amine in three lung adenocarcinoma cells, six tissues, and serum from healthy controls and patients with benign lung disease as well as in lung cancer patients. The panels demonstrate particular implications for the diagnosis of physiopathological states, which need to be further validated in a large cohort in the future. The PMPs demonstrate promising potential for the detailed study of the biochemical landscape and biomarker discovery.

## Data Availability

The original contributions presented in the study are included in the article/Supplementary Material, further inquiries can be directed to the corresponding authors.
