# Supplementary material for: Profiling of amines in biological samples using polythioester-functionalized magnetic nanoprobe
Source: Front Bioeng Biotechnol. 2023 Jan 4;10:1103995. doi: 10.3389/fbioe.2022.1103995 (PMC9846243; doi:10.3389/fbioe.2022.1103995)
Supplement: Supplementary file 1 [file DataSheet1.docx]

Supplementary Material

# Mass spectrometric analysis

The reaction condition optimization data were acquired by an LTQ XL^TM^ linear ion trap mass spectrometer (Thermo Scientific,). Thermo Scientific^TM^ Xcalibur^TM^ software for method setup, data acquisition, data processing, and reporting. In the negative ion mode, the capillary voltage was 3.6 kV, the capillary temperature was 200 °C, and the injection flow rate was 8 μL/min.

Mass spectrometry data for the polymer was collected via Ultraflex MALDI-TOF MS (Bruker Daltonics, Billerica, MA, USA).The acquisition was performed in positive ion mode with a range of 300-1800 Da and 2, 5-dihydroxybenzoic acid (DHB) as the matrix (20mg DHB dissolved in 1mL 50% ACN containing 1‰ trifluoroacetic acid). DHB (0.01mg) and polymer (0.002 mg) were spotted on MTP 384 ground steel plate and dried at room temperature.

Mass spectrometry data of biological samples were acquired by a 7.0 T Apex-ultraTM hybrid Qh-FTICR mass spectrometry, and all mass spectra were acquired using ApexControl 3.0.0 (Bruker Daltonics, Billerica, MA, USA) software. Mass spectrometry data acquisition was performed in negative ion mode, the capillary voltage was 2500 V, the drying gas flow rate was set to 5.0 L/min, the drying gas temperature was 200 °C, the nebulizing gas was 1.0 bar, the acquisition range was 100-600 Da, and the acquisition size was 1 M, the resolution was 66000 (m/z at 400), and the acquisition was 20 scans. The injection flow rate was 300 μL/h.

# Biological sample pre-processing

## Tissues

50 mg of heart, liver, spleen, lung, kidney, and brain tissue of mice, homogenized using a high-speed tissue grinder. After homogenizing, add water to make the mixing volume 100 μL and add 900 μL of ice methanol containing isotope-labeled amino acids (15.97 μM L-Glu-^13^C_5_, 7.5 μM L-Val-^13^C_5_,^15^N, 3.33 μM L-Tyr-^13^C_6_, 36 μM L-Arg-^13^C_6_, 6.67 μM L-Gly-^13^C_2_), sonicate for 1 min, vortex mixing for 30 s. Precipitate proteins at -20 °C overnight. Then, centrifuge (4 °C, 15,000× g) for 30 min, take 100 μL of the supernatant, and concentrate in vacuo.

## Lung adenocarcinoma cell

2× 10^6^ HCC827, NCI-H1650, NCI-H2228 cells, added 50 μL of water, let stand for 5 min, centrifuge (4 °C, 15000×g) for 30 min, take the supernatant, repeat this operation 3 times. Add 850 μL of ice methanol containing isotope-labeled amino acids (15.97 μM L-Glu-^13^C_5_, 7.5 μM L-Val-^13^C_5_, ^15^N, 3.33 μM L-Tyr-^13^C_6_, 36 μM L-Arg-^13^C_6_, 6.67 μM L-Gly-^13^C_2_) and vortex to mix well and precipitate the protein overnight at -20 °C. Then, centrifuge (4 °C, 15000×g) for 30 min, take the supernatant and concentrate in vacuo.

## Serum

100 μL serum, add 900 μL of ice methanol containing isotope-labeled amino acids (15.97 μM L-Glu-^13^C_5_, 7.5 μM L-Val-^13^C_5_,^15^N, 3.33 μM L-Tyr-^13^C_6_, 36 μM L-Arg-^13^C_6_, 6.67 μM L-Gly-^13^C_2_), vortex for 30 s, and precipitate proteins overnight at -20 °C. Then, centrifuge (4 °C, 15,000× g) for 30 min, take 300 μL of the supernatant and concentrate in vacuo.


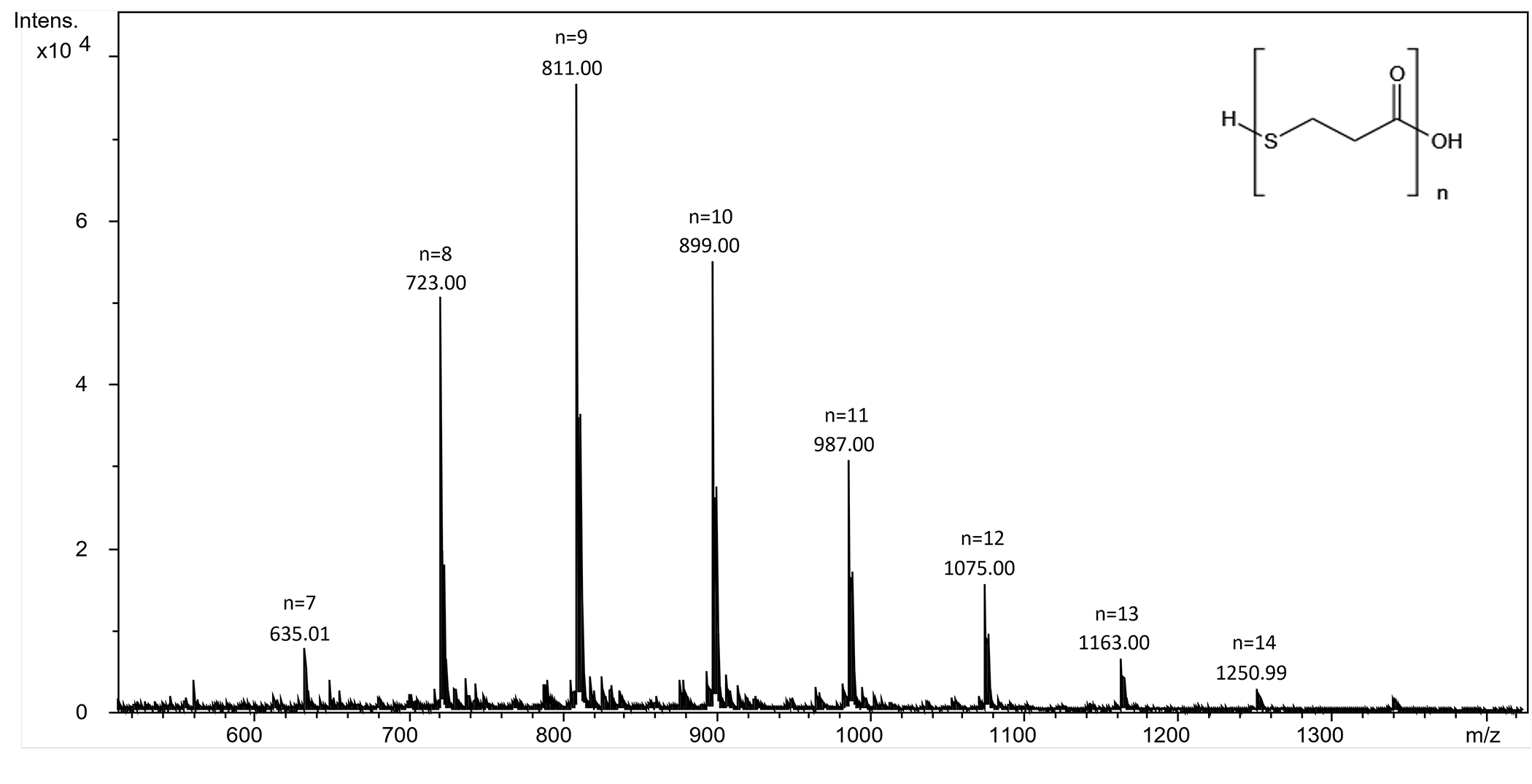


**Supplementary Figure 1.** MALDI-TOF mass spectrum of polythioesters synthesized with mercaptopropionic acid.

**Supplementary Table 1.** Structural assignments for the peaks observed in the MALDI-TOF mass spectrum of polythioesters synthesized with mercaptopropionic acid.

| Examples | m/z | Proposed oligomer structure |
| --- | --- | --- |
| 635.01 (n=7)  723.00 (n=8)  811.00 (n=9)  899.00 (n=10)  987.00 (n=11)  1075.00 (n=12)  1163.00 (n=13)  1250.99 (n=14) | n×87.998+1.008+17.003+1.007 | H–[C_3_H_4_SO]_n_–OH +H^+^ |


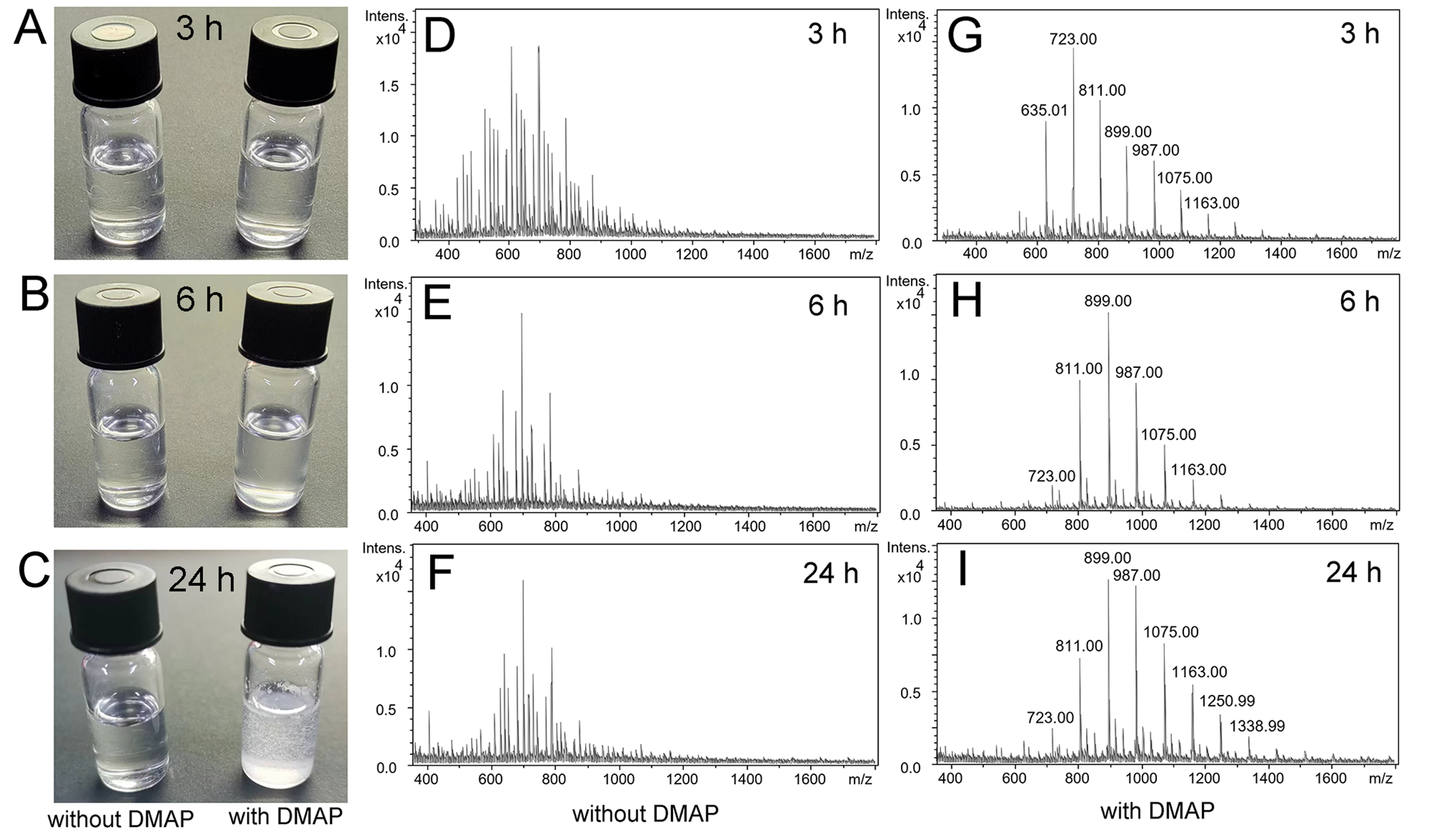


**Supplementary Figure 2**. The photographs and MALDI-TOF mass spectra of the reaction products of mercaptopropionic acid and EDC with or without the addition of DMAP.


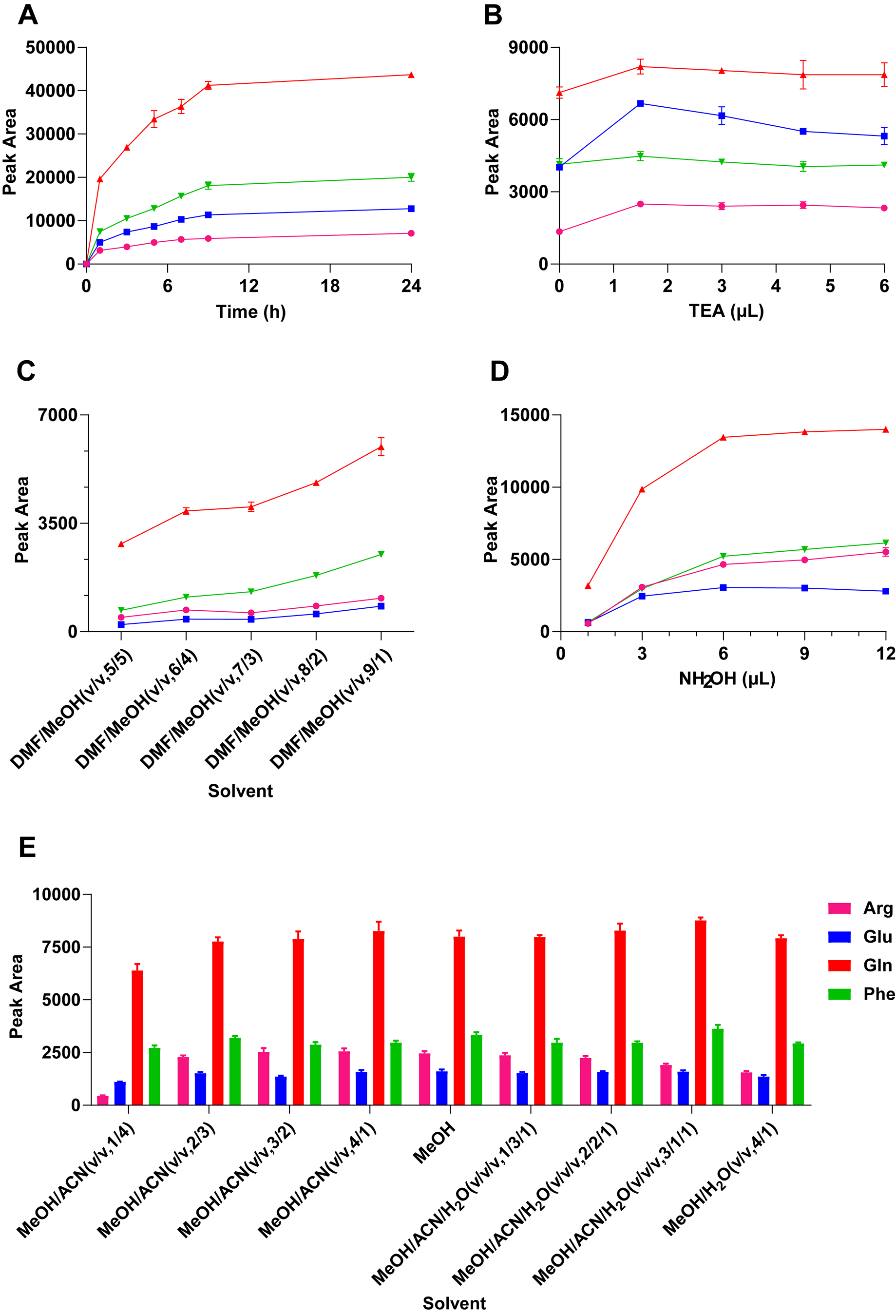


**Supplementary Figure 3.** Optimization of reaction conditions. Reaction time of carboxyl group activation (A), addition of TEA (B), solvent conditions for capture (C), NH_2_OH concentration (D), and solvent conditions for release (J).

**Supplementary Table 2.** Theoretical and experimental m/z values, mass error, linearity, LOQ, LOD, precision and recovery of amines in ESI-FTICR MS negative ion mode.

|  | Theoretical  m/z  [M-H]^-^ | Observed  m/z  [M-H]^-^ | Error  (ppm) | Linearity | | | LOQ  (μM) | LOD  (μM) | Precision (RSD, %) | | Recovery (%) | | |
| --- | --- | --- | --- | --- | --- | --- | --- | --- | --- | --- | --- | --- | --- |
|  |  |  |  | Calibration curve | R^2^ | Linear ranges  (μM) |  |  | Intra-day | Inter-day | R1 | R2 | R3 |
| Glycine | 162.0230 | 162.0230 | 0.00 | y = 0.8994x + 0.1793 | 0.9998 | 0.3975 - 79.5 | 0.0795 | 0.0795 | 1.57 | 4.42 | 102.57 | 107.53 | 106.35 |
| Alanine | 176.0387 | 176.0387 | 0.00 | y = 0.5925x + 0.9594 | 0.999 | 1.215 - 121.5 | 0.6075 | 0.6075 | 0.34 | 3.30 | 96.26 | 92.23 | 98.28 |
| Serine | 192.0336 | 192.0336 | 0.00 | y = 1.6703x + 3.5233 | 0.9964 | 0.1965 - 19.65 | 0.1965 | 0.1965 | 2.58 | 2.45 | 85.71 | 85.98 | 84.35 |
| Proline | 202.0543 | 202.0543 | 0.00 | y = 0.135x + 0.0043 | 0.9995 | 0.708 - 3.54 | 0.0708 | 0.0354 | 2.30 | 2.79 | 96.00 | 89.48 | 75.96 |
|  |  |  |  | y = 0.0812x + 0.1563 | 0.9953 | 3.54 - 70.8 |  |  |  |  |  |  |  |
| Valine | 204.0700 | 204.0700 | 0.00 | y = 0.4466x + 0.0295 | 0.9989 | 0.065 - 64.95 | 0.0325 | 0.0325 | 1.53 | 3.84 | 104.17 | 107.78 | 110.84 |
| Threonine | 206.0493 | 206.0492 | -0.49 | y = 0.274x + 0.0039 | 0.9993 | 0.2145 - 21.45 | 0.2145 | 0.2145 | 1.63 | 4.20 | 95.85 | 96.83 | 101.79 |
| Taurine | 212.0057 | 212.0057 | 0.00 | y = 1.6334x - 0.7803 | 0.997 | 0.0185 - 73.8 | 0.0185 | 0.0037 | 3.00 | 2.28 | 90.15 | 98.62 | 102.47 |
| Leucine/Isoleucine | 218.0856 | 218.0856 | 0.00 | y = 0.9434x - 0.0086 | 0.9985 | 0.0162 - 0.81 | 0.0162 | 0.0032 | 2.64 | 4.24 | 102.81 | 99.26 | 104.84 |
|  |  |  |  | y = 0.8471x + 0.0663 | 0.9989 | 0.81 - 16.2 |  |  |  |  |  |  |  |
| Glutamine | 233.0602 | 233.0601 | 0.43 | y = 0.2348x + 0.0153 | 0.9979 | 0.159 - 159 | 0.0795 | 0.0795 | 1.43 | 7.36 | 107.01 | 104.06 | 115.32 |
| Methionine | 236.0421 | 236.0420 | 0.42 | y = 3.5814x + 0.0766 | 0.9981 | 0.0375 - 7.5 | 0.0375 | 0.0375 | 1.21 | 5.12 | 98.23 | 95.06 | 108.49 |
| Phenylalanine | 252.0700 | 252.0700 | 0.00 | y = 4.102x - 0.8785 | 0.9951 | 0.0952 - 9.52 | 0.0095 | 0.0095 | 2.04 | 2.86 | 90.53 | 90.86 | 93.41 |
| Tyrosine | 268.0649 | 268.0649 | 0.00 | y = 0.8972x - 0.0718 | 0.9995 | 0.0975 - 9.75 | 0.0975 | 0.0195 | 1.17 | 3.63 | 96.30 | 95.40 | 96.60 |
| Tryptophan | 291.0809 | 291.0808 | 0.34 | y = 0.6434x - 0.0174 | 0.9986 | 0.096 - 9.6 | 0.0960 | 0.0192 | 2.63 | 4.36 | 97.67 | 99.71 | 98.47 |
| N2-Acetyl-L-Lysine | 275.1071 | 275.1071 | 0.00 | y = 3.7666x - 0.0021 | 0.9999 | 0.015 - 3.75 | 0.0150 | 0.015 | 4.55 | 14.88 | 103.80 | 105.26 | 100.00 |
| Citrulline | 262.0867 | 262.0867 | 0.00 | y = 1.0082x + 0.1942 | 0.9998 | 0.075 - 15 | 0.075 | 0.075 | 0.82 | 1.51 | 95.83 | 92.01 | 95.07 |
| Hydroxyproline | 218.0493 | 218.0493 | 0.00 | y = 0.0814x + 0.007 | 0.999 | 0.15 - 15 | 0.075 | 0.015 | 1.38 | 6.51 | 111.59 | 106.53 | 112.64 |
| Asparagine | 219.0445 | 219.0445 | 0.00 | y = 0.5795x - 0.2418 | 0.9952 | 0.27 - 13.5 | 0.1350 | 0.027 | 2.10 | 3.19 | 90.37 | 88.12 | 91.62 |
| Arginine | 261.1027 | 261.1027 | 0.00 | y = 1.5153x - 0.0155 | 0.9999 | 0.5287 - 52.87 | 0.5287 | 0.0529 | 3.62 | 2.69 | 100.00 | 103.21 | 112.05 |
| Lysine | 321.0948 | 321.0948 | 0.00 | y = 1.7238x - 1.0679 | 0.9943 | 0.2258 - 45.15 | 0.2258 | 0.0226 | 3.78 | 6.66 | 74.30 | 76.92 | 81.12 |
| Histidine | 242.0605 | 242.0604 | 0.41 | y = 0.4843x + 0.0345 | 0.9946 | 0.135 - 13.5 | 0.1350 | 0.0135 | 4.42 | 12.47 | 108.77 | 110.41 | 108.98 |
| Ornithine | 307.0792 | 307.0792 | 0.00 | y = 1.6089x - 0.3943 | 0.994 | 0.0825 - 16.5 | 0.0825 | 0.0165 | 2.48 | 4.32 | 76.17 | 79.27 | 83.23 |
| Aspartic acid | 220.0285 | 220.0285 | 0.00 | y = 3.6698x + 0.2282 | 0.9915 | 0.075 - 7.5 | 0.0750 | 0.075 | 3.97 | 16.71 | 90.07 | 98.47 | 108.88 |
| Glutamic acid | 234.0442 | 234.0441 | 0.43 | y = 3.148x + 0.1337 | 0.9968 | 0.0818 - 16.35 | 0.0818 | 0.0818 | 5.24 | 10.58 | 84.36 | 85.79 | 106.07 |
| GABA | 190.0543 | 190.0543 | 0.00 | y = 1.3199x - 0.0302 | 0.9973 | 0.0375 - 3.75 | 0.0375 | 0.0375 | 2.02 | 3.69 | 85.96 | 82.97 | 86.45 |
| Alanylglutamine | 304.0973 | 304.0973 | 0.00 | y = 0.5083x - 0.0173 | 0.9975 | 0.045 - 4.5 | 0.0450 | 0.0225 | 3.59 | 2.20 | 85.51 | 96.67 | 98.69 |

**Supplementary Table 3.** **The concentration of amine in the cells.**

| AA（nmol/10^6^） | lung adenocarcinoma cells | | |
| --- | --- | --- | --- |
|  | NCI-H1650（n=3）  Mean [95% CI] | NCI-H2228（n=3）  Mean [95% CI] | HCC827（n=3）  Mean [95% CI] |
| Glycine | 6.754 [6.651-6.856] | 3.641 [3.59-3.693] | 5.379 [5.282-5.476] |
| Alanine | 2.109 [1.677-2.541] | 1.197 [1.024-1.37] | 2.5 [2.164-2.837] |
| Serine | 0.2009 [0.1196-0.2822] |  | 0.3613 [0.261-0.4616] |
| Proline | 3.427 [2.927-3.928] | 2.947 [2.884-3.01] | 2.535 [2.258-2.813] |
| Valine | 0.6564 [0.6173-0.6954] | 0.8027 [0.7706-0.8348] | 1.191 [1.103-1.279] |
| Threonine | 0.5889 [0.4891-0.6886] | 0.5979 [0.5448-0.6509] | 0.7489 [0.639-0.8587] |
| Taurine | 5.573 [5.017-6.128] | 1.461 [1.279-1.643] | 2.024 [1.891-2.157] |
| Leucine/Isoleucine | 2.403 [2.101-2.705] | 1.785 [1.667-1.902] | 2.31 [2.065-2.554] |
| Glutamine | 1.08 [0.7196-1.44] | 1.056 [1.011-1.102] | 0.3266 [0.2285-0.4247] |
| Methionine | 0.1137 [0.09695-0.1305] | 0.08082 [0.07919-0.08245] | 0.1226 [0.1166-0.1285] |
| Phenylalanine | 0.4242 [0.3932-0.4551] | 0.3343 [0.3121-0.3565] | 0.4838 [0.4764-0.4913] |
| Tyrosine | 0.3936 [0.381-0.4063] | 0.3215 [0.3054-0.3375] | 0.4347 [0.4286-0.4408] |
| Tryptophan | 0.1178 [0.1122-0.1234] |  | 0.1005 [0.08693-0.114] |
| N-Acetyl-L-lysine | 0.008885 [0.007891-0.009878] | 0.01252 [0.01145-0.01358] | 0.01221 [0.01167-0.01275] |
| Citrulline | 0.0730 [0.06036-0.08581] | 0.0506 [0.0303-0.07103] | 0.0721 [0.06193-0.08227] |
| Hydroxyproline | 0.3588 [0.2528-0.4648] | 0.3822 [0.3581-0.4064] | 0.2543 [0.1974-0.3111] |
| Asparagine | 0.1924 [0.1535-0.2314] | 0.2428 [0.2363-0.2493] | 0.1745 [0.1577-0.1913] |
| Arginine | 0.8317 [0.7921-0.8712] | 0.9185 [0.7973-1.04] | 1.461 [1.417-1.505] |
| Lysine | 0.5301 [0.5055-0.5548] | 0.7666 [0.7171-0.816] | 0.7747 [0.7608-0.7885] |
| Histidine | 0.1546 [0.08457-0.2246] | 0.182 [0.1782-0.1858] | 0.1402 [0.1075-0.1728] |
| Ornithine | 0.1409 [0.1381-0.1437] | 0.2587 [0.254-0.2634] | 0.1546 [0.1517-0.1576] |
| N-Ac-Orn | 0.05299 [0.04806-0.05793] | 0.1224 [0.1037-0.141] | 0.04969 [0.0495-0.04988] |
| Aspartic acid | 0.241 [0.03052-0.5126] | 0.3171 [0.2389-0.3952] | 1.016 [0.6847-1.347] |
| Glutamic acid | 2.232 [1.568-2.897] | 2.621 [2.082-3.159] | 2.431 [1.674-3.189] |
| Cystathionine | 0.02372 [0.01806-0.02937] | 0.006256 [0.003847-0.008665] | 0.01389 [0.01206-0.01572] |
| 5-Hydroxylysine | 0.005372 [0.004361-0.006384] | 0.009595 [0.0094-0.009789] | 0.007202 [0.005809-0.008594] |


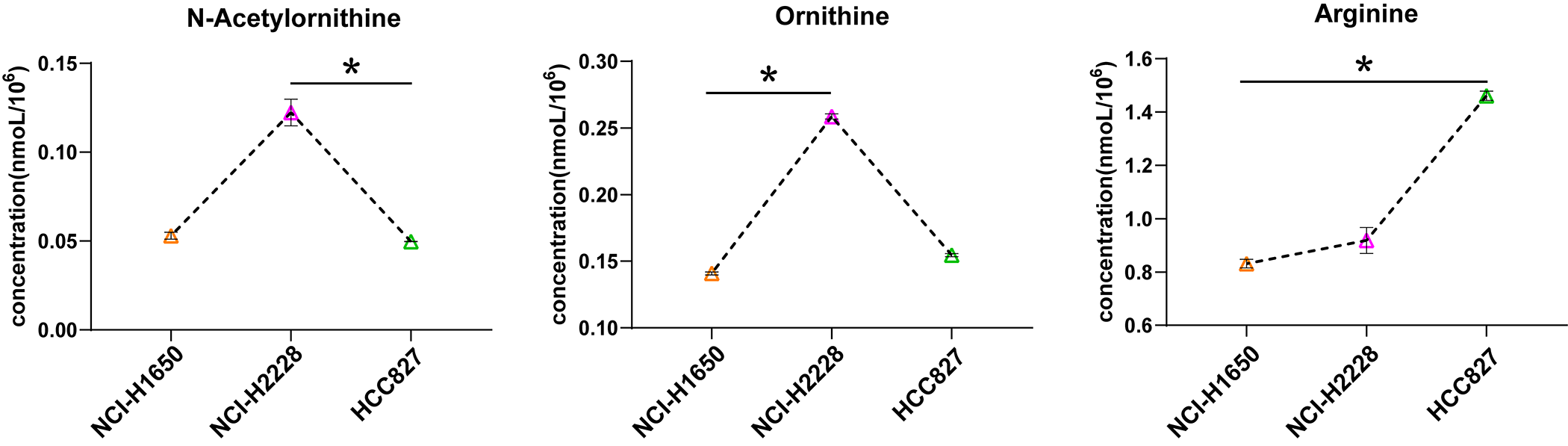


**Supplementary Figure 4.** Differences in concentrations of N-Acetylornithine, Ornithine, and Arginine in lung adenocarcinoma cells. Data are described as mean ± standard deviation. (*, p<0.05)

**Supplementary Table 4.** Kruskal-Wallis test of amines in lung adenocarcinoma cells.

|  | NCI-H1650  *vs.*  NCI-H2228 | NCI-H1650  *vs.*  HCC827 | NCI-H2228  *vs.*  HCC827 |
| --- | --- | --- | --- |
| Glycine | **0.0219** | 0.5391 | 0.5391 |
| Alanine | 0.5391 | 0.5391 | **0.0219** |
| Proline | 0.5391 | **0.0219** | 0.5391 |
| Valine | 0.5391 | **0.0219** | 0.5391 |
| Threonine | >0.9999 | 0.1107 | 0.1579 |
| Taurine | **0.0219** | 0.5391 | 0.5391 |
| Leucine/Isoleucine | 0.0512 | >0.9999 | 0.3032 |
| Glutamine | >0.9999 | 0.076 | 0.2209 |
| Methionine | 0.5391 | 0.5391 | **0.0219** |
| Phenylalanine | 0.5391 | 0.5391 | **0.0219** |
| Tyrosine | 0.5391 | 0.5391 | 0.0219 |
| N-Acetyl-L-lysine | 0.0512 | 0.3032 | >0.9999 |
| Citrulline | 0.1107 | >0.9999 | 0.1579 |
| Hydroxyproline | >0.9999 | 0.2209 | 0.076 |
| Asparagine | 0.3032 | >0.9999 | 0.0512 |
| Arginine | 0.5391 | **0.0219** | 0.5391 |
| Lysine | 0.2209 | 0.076 | >0.9999 |
| Histidine | 0.2209 | >0.9999 | 0.076 |
| Ornithine | **0.0219** | 0.5391 | 0.5391 |
| N-Acetylornithine | 0.5391 | 0.5391 | **0.0219** |
| Aspartic acid | >0.9999 | 0.0512 | 0.3032 |
| Glutamic acid | 0.3032 | >0.9999 | 0.8902 |
| Cystathionine | **0.0219** | 0.5391 | 0.5391 |
| 5-Hydroxylysine | **0.0219** | 0.5391 | 0.5391 |

**Supplementary Table 5.** **The concentration of amines in tissues.**

| AA（nmoL/mg） | Tissue | | | | | |
| --- | --- | --- | --- | --- | --- | --- |
|  | spleen（n=5） | liver（n=5） | heart（n=5） | kidney（n=5） | lung（n=5） | brain（n=5） |
|  | Mean [95% CI] | Mean [95% CI] | Mean [95% CI] | Mean [95% CI] | Mean [95% CI] | Mean [95% CI] |
| Glycine | 2.268 [2.058-2.478] | 2.644 [2.129-3.160] | 0.5082 [0.4359-0.5805] | 5.306 [4.505-6.107] | 4.100 [3.818-4.382] | 1.117 [0.9976-1.236] |
| Alanine | 1.422 [1.153-1.692] | 3.483 [2.343-4.622] | 1.663 [1.411-1.916] | 2.882 [2.338-3.426] | 1.884 [1.726-2.041] | 1.034 [0.9175-1.151] |
| Proline | 0.2684 [0.1859-0.3509] | 0.3155 [0.1698-0.4611] | 0.04247 [0.02896-0.05597] | 0.6339 [0.4251-0.8426] | 0.1897 [0.1489-0.2305] | 0.05956 [0.04194-0.07718] |
| Valine | 0.4640 [0.3795-0.5484] | 0.6639 [0.4607-0.8670] | 0.1164 [0.07672-0.1561] | 0.6890 [0.5217-0.8563] | 0.3304 [0.2510-0.4099] | 0.08355 [0.07570-0.09141] |
| Threonine | 0.2809 [0.2432-0.3187] | 0.2686 [0.1923-0.3448] | 0.1124 [0.09893-0.1258] | 0.4210 [0.2131-0.6289] | 0.2206 [0.1914-0.2498] | 0.2004 [0.1705-0.2303] |
| Taurine | 9.235 [7.373-11.10] | 7.437 [4.137-10.74] | 12.88 [10.10-15.66] | 4.362 [3.176-5.547] | 4.53 [3.607-5.453] | 5.913 [4.567-7.259] |
| Leucine/Isoleucine | 1.452 [1.167-1.737] | 1.494 [0.9643-2.023] | 0.4245 [0.3016-0.5475] | 1.475 [1.115-1.834] | 0.6190 [0.4657-0.7724] | 0.1518 [0.1285-0.1752] |
| Glutamine | 1.624 [1.579-1.669] | 2.478 [1.526-3.431] | 3.764 [3.148-4.379] | 1.546 [1.268-1.824] | 1.640 [1.212-2.067] | 4.145 [3.278-5.011] |
| Methionine | 0.1590 [0.1374-0.1806] | 0.1516 [0.1097-0.1934] | 0.04970 [0.04132-0.05808] | 0.2095 [0.1810-0.2381] | 0.09397 [0.08607-0.1019] | 0.03813 [0.03220-0.04406] |
| Phenylalanine | 0.2412 [0.1955-0.2869] | 0.1710 [0.1304-0.2115] | 0.09821 [0.08369-0.1127] | 0.2156 [0.1238-0.3074] | 0.1307 [0.1218-0.1397] | 0.08340 [0.07787-0.08892] |
| Tyrosine | 0.1908 [0.1541-0.2275] | 0.1912 [0.1050-0.2774] | 0.05554 [0.04558-0.06549] | 0.3261 [0.2062-0.4460] | 0.09172 [0.07922-0.1042] | 0.04466 [0.03751-0.05182] |
| Tryptophan | 0.07484 [0.06258-0.08710] | 0.03521 [0.02749-0.04292] | 0.03809 [0.03255-0.04363] | 0.08509 [0.06757-0.1026] | 0.04439 [0.04040-0.04837] | 0.02778 [0.02128-0.03428] |
| N-Acetyl-L-Lysine | 0.01129 [0.008912-0.01367] | 0.01672 [0.01127-0.02216] | 0.003501 [0.003021-[0.003981] | 0.009382 [0.008417-0.01035] | 0.005481 [0.003732-[0.007231] | 0.007108 [0.005878-0.008339] |
| Citrulline | 0.1678 [0.1143-0.2212] | 0.1965 [0.1623-0.2306] | 0.1688 [0.1396-0.1981] | 0.07834 [0.05303-0.1037] | 0.1093 [0.07675-0.1419] | 0.05952 [0.04344-0.07561] |
| Hydroxyproline | 0.03756 [0.02451-0.05061] | 0.03203 [0.01839-0.04568] | 0.01231 [0.003353-0.02127] | 0.02577 [0.0148-0.03675] | 0.01537 [0.009553-0.02119] | 0.008612 [0.002829-0.01439] |
| Asparagine | 0.2459 [0.2252-0.2667] | 0.192 [0.1568-0.2271] | 0.1818 [0.1629-0.2007] | 0.2649 [0.1851-0.3447] | 0.1567 [0.151-0.1624] | 0.1536 [0.1437-0.1636] |
| Arginine | 0.02698 [0.0113-0.04267] |  | 0.0325 [0.01506-0.04994] | 0.09376 [0.02102-0.1665] | 0.01952 [0.01172-0.02733] |  |
| Lysine | 0.3299 [0.3211-0.3387] | 0.3608 [0.3234-0.3982] | 0.3147 [0.3008-0.3286] | 0.3095 [0.2865-0.3325] | 0.2841 [0.2764-0.2919] | 0.2831 [0.274-0.2921] |
| Histidine | 0.1035 [0.08963-0.1173] | 0.2184 [0.1455-0.2913] | 0.07235 [0.04964-0.09506] | 0.09808 [0.07665-0.1195] | 0.03024 [0.0171-0.04337] | 0.01001 [0.00000-0.02505] |
| Ornithine | 0.1211 [0.1163-0.1258] | 0.1813 [0.16-0.2027] | 0.1115 [0.1095-0.1134] | 0.1199 [0.1173-0.1225] | 0.1159 [0.112-0.1198] | 0.1066 [0.1036-0.1096] |
| N2-AcetylOrnithine | 0.03402 [0.03016-0.03787] | 0.01958 [0.01378-0.02538] | 0.02527 [0.02308-0.02745] | 0.03106 [0.0266-0.03551] | 0.01564 [0.01315-0.01814] | 0.08893 [0.07334-0.1045] |
| Aspartic acid | 0.4622 [0.376-0.5483] | 0.01319 [0.003983-0.02239] | 0.0959 [0.02183-0.17] | 0.2529 [0.09265-0.4131] | 0.08845 [0.04084-0.1361] | 0.5474 [0.3142-0.7805] |
| Glutamic acid | 1.132 [0.9908-1.273] | 0.3559 [0.1794-0.5324] | 0.7261 [0.511-0.9411] | 1.23 [1.14-1.319] | 0.2943 [0.2636-0.3251] | 1.759 [1.569-1.95] |
| GABA | 0.1214 [0.08701-0.1557] | 0.104 [0.08504-0.123] | 0.04235 [0.03439-0.0503] | 0.1119 [0.0544-0.1694] | 0.04517 [0.03924-0.0511] | 2.27 [1.98-2.561] |
| Alanylglutamine | 0.05047 [0.04521-0.05573] | 0.06047 [0.05084-0.0701] | 0.07478 [0.06761-0.08196] | 0.0697 [0.04876-0.09064] | 0.05851 [0.05193-0.06509] | 0.09598 [0.08449-0.1075] |
| Cystathionine | 0.03823 [0.03229-0.04416] | 0.03898 [0.03339-0.04457] | 0.01908 [0.01829-0.01987] | 0.08394 [0.0588-0.1091] | 0.0439 [0.04103-0.04677] | 0.02296 [0.01912-0.0268] |

**Supplementary Table 6.** Kruskal-Wallis test of amines in mouse tissues.

|  | Spleen  *vs.*  liver | spleen  *vs.*  heart | spleen  *vs.*  kidney | spleen  *vs.*  lung | spleen  *vs.*  brain | liver  *vs.*  heart | liver  *vs.*  kidney | liver  *vs.*  lung | liver  *vs.*  brain | heart  *vs.*  kidney | heart  *vs.*  lung | heart  *vs.*  brain | kidney  *vs.*  lung | kidney  *vs.*  brain | lung  *vs.*  brain |
| --- | --- | --- | --- | --- | --- | --- | --- | --- | --- | --- | --- | --- | --- | --- | --- |
| Glycine | >0.9999 | 0.7229 | 0.1979 | >0.9999 | >0.9999 | 0.1788 | 0.7862 | >0.9999 | >0.9999 | **0.0001** | **0.0043** | >0.9999 | >0.9999 | **0.0056** | **0.095** |
| Alanine | **0.0208** | >0.9999 | 0.0762 | >0.9999 | >0.9999 | 0.2187 | >0.9999 | >0.9999 | **0.0003** | 0.6091 | >0.9999 | >0.9999 | >0.9999 | **0.0018** | 0.1614 |
| Proline | >0.9999 | 0.0543 | >0.9999 | >0.9999 | 0.664 | **0.0236** | >0.9999 | >0.9999 | 0.3545 | **0.0001** | 0.5582 | >0.9999 | 0.2933 | **0.0065** | >0.9999 |
| Valine | >0.9999 | 0.6091 | >0.9999 | >0.9999 | 0.1455 | **0.0208** | >0.9999 | 0.6091 | **0.0028** | 0.0143 | >0.9999 | >0.9999 | 0.4671 | **0.0018** | >0.9999 |
| Threonine | >0.9999 | **0.0074** | >0.9999 | >0.9999 | 0.2933 | 0.0762 | >0.9999 | >0.9999 | >0.9999 | **0.0004** | >0.9999 | >0.9999 | 0.2663 | **0.034** | >0.9999 |
| Taurine | >0.9999 | >0.9999 | 0.0681 | 0.0762 | >0.9999 | >0.9999 | 0.664 | 0.7229 | >0.9999 | **0.0021** | **0.0024** | 0.1979 | >0.9999 | >0.9999 | >0.9999 |
| Leucine/Isoleucine | >0.9999 | 0.131 | >0.9999 | >0.9999 | **0.0056** | 0.0762 | >0.9999 | 0.7229 | **0.0028** | 0.1614 | >0.9999 | >0.9999 | >0.9999 | **0.0074** | >0.9999 |
| Glutamine | >0.9999 | 0.1788 | >0.9999 | >0.9999 | 0.0681 | >0.9999 | 0.5109 | >0.9999 | >0.9999 | **0.0267** | 0.0681 | >0.9999 | >0.9999 | **0.0085** | **0.0236** |
| Methionine | >0.9999 | 0.2933 | >0.9999 | >0.9999 | 0.0236 | 0.2663 | >0.9999 | >0.9999 | **0.0208** | **0.0074** | >0.9999 | >0.9999 | 0.1614 | **0.0002** | >0.9999 |
| Phenylalanine | >0.9999 | **0.0097** | >0.9999 | 0.2933 | **0.0003** | 0.7229 | >0.9999 | >0.9999 | 0.0762 | 0.1179 | >0.9999 | >0.9999 | >0.9999 | **0.0074** | 0.854 |
| Tyrosine | >0.9999 | 0.2933 | >0.9999 | >0.9999 | **0.0382** | 0.2933 | >0.9999 | >0.9999 | **0.0382** | **0.0049** | >0.9999 | >0.9999 | 0.2187 | **0.0003** | >0.9999 |
| Tryptophan | 0.1179 | 0.2933 | >0.9999 | >0.9999 | **0.0056** | >0.9999 | **0.0267** | >0.9999 | >0.9999 | 0.0762 | >0.9999 | >0.9999 | >0.9999 | **0.0009** | 0.3545 |
| N-Acetyl-L-Lysine | >0.9999 | **0.0049** | >0.9999 | 0.1614 | 0.7862 | **0.0002** | >0.9999 | **0.0125** | 0.095 | 0.0762 | >0.9999 | >0.9999 | >0.9999 | >0.9999 | >0.9999 |
| Citrulline | >0.9999 | >0.9999 | 0.2663 | >0.9999 | **0.034** | >0.9999 | **0.0162** | 0.2187 | **0.0012** | 0.1788 | >0.9999 | **0.0208** | >0.9999 | >0.9999 | >0.9999 |
| Hydroxyproline | >0.9999 | **0.0382** | >0.9999 | 0.3545 | **0.0097** | 0.1788 | >0.9999 | >0.9999 | 0.0543 | 0.7229 | >0.9999 | >0.9999 | >0.9999 | 0.2663 | >0.9999 |
| Asparagine | >0.9999 | 0.7862 | >0.9999 | **0.0097** | **0.0043** | >0.9999 | >0.9999 | >0.9999 | >0.9999 | 0.9267 | >0.9999 | >0.9999 | **0.0125** | **0.0056** | >0.9999 |
| Arginine | 0.2241 | >0.9999 | >0.9999 | >0.9999 | 0.2241 | 0.0684 | **0.0008** | >0.9999 | >0.9999 | >0.9999 | >0.9999 | 0.0684 | 0.2604 | **0.0008** | >0.9999 |
| Lysine | >0.9999 | >0.9999 | >0.9999 | **0.0382** | **0.0236** | >0.9999 | 0.854 | **0.0056** | **0.0032** | >0.9999 | 0.7862 | 0.5582 | >0.9999 | >0.9999 | >0.9999 |
| Histidine | >0.9999 | >0.9999 | >0.9999 | 0.2931 | **0.0382** | 0.1787 | >0.9999 | **0.0032** | **0.0002** | >0.9999 | >0.9999 | 0.9262 | 0.4263 | 0.0608 | >0.9999 |
| Ornithine | >0.9999 | 0.3226 | >0.9999 | >0.9999 | **0.0236** | **0.0049** | >0.9999 | 0.1788 | **0.0001** | 0.7229 | >0.9999 | >0.9999 | >0.9999 | 0.0681 | 0.7862 |
| Aspartic acid | **0.0021** | 0.2924 | >0.9999 | 0.1306 | >0.9999 | >0.9999 | 0.0947 | >0.9999 | **0.0007** | >0.9999 | >0.9999 | 0.145 | >0.9999 | >0.9999 | 0.0606 |
| Glutamic acid | 0.2933 | >0.9999 | >0.9999 | 0.1614 | >0.9999 | >0.9999 | 0.0762 | >0.9999 | **0.0014** | >0.9999 | >0.9999 | 0.095 | **0.0382** | >0.9999 | **0.0005** |
| GABA | >0.9999 | 0.1179 | >0.9999 | 0.1979 | >0.9999 | 0.4671 | >0.9999 | 0.7229 | 0.7229 | 0.4266 | >0.9999 | **0.0005** | 0.664 | 0.7862 | **0.0012** |
| Alanylglutamine | >0.9999 | **0.043** | 0.4671 | >0.9999 | **0.0003** | >0.9999 | >0.9999 | >0.9999 | 0.1059 | >0.9999 | >0.9999 | >0.9999 | >0.9999 | 0.5582 | 0.0851 |
| N2-AcetylOrnithine | 0.2933 | >0.9999 | >0.9999 | **0.0208** | >0.9999 | >0.9999 | 0.7862 | >0.9999 | **0.0065** | >0.9999 | >0.9999 | 0.0762 | 0.0762 | >0.9999 | **0.0002** |
| Cystathionine | >0.9999 | 0.4266 | 0.5109 | >0.9999 | >0.9999 | 0.3545 | 0.6091 | >0.9999 | >0.9999 | **0.0002** | **0.0301** | >0.9999 | >0.9999 | **0.0024** | 0.1614 |


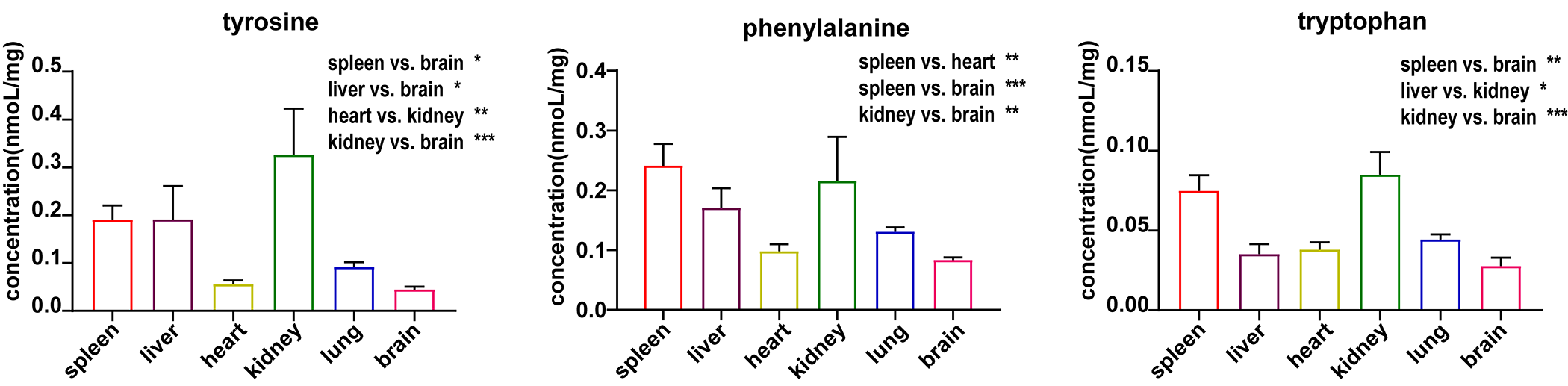


**Supplementary Figure 5.** Differences in the concentrations of tyrosine, phenylalanine, and tryptophan in organ tissues. Data are described as mean ± standard deviation. (*, p<0.05, **, p<0.01, ***, p<0.001)

**Supplementary Table 7.** Clinical Information of Serum Samples.

|  | Subjects | | Age（yr） | |
| --- | --- | --- | --- | --- |
|  | Total | Female/Male | Range | mean±SD |
| HCs | 29 | 14/15 | 37-69 | 49.34±10.07 |
| BLDs | 37 | 17/20 | 37-66 | 50.16±9.329 |
| LCs | 37 | 17/20 | 37-69 | 52.30±10.75 |


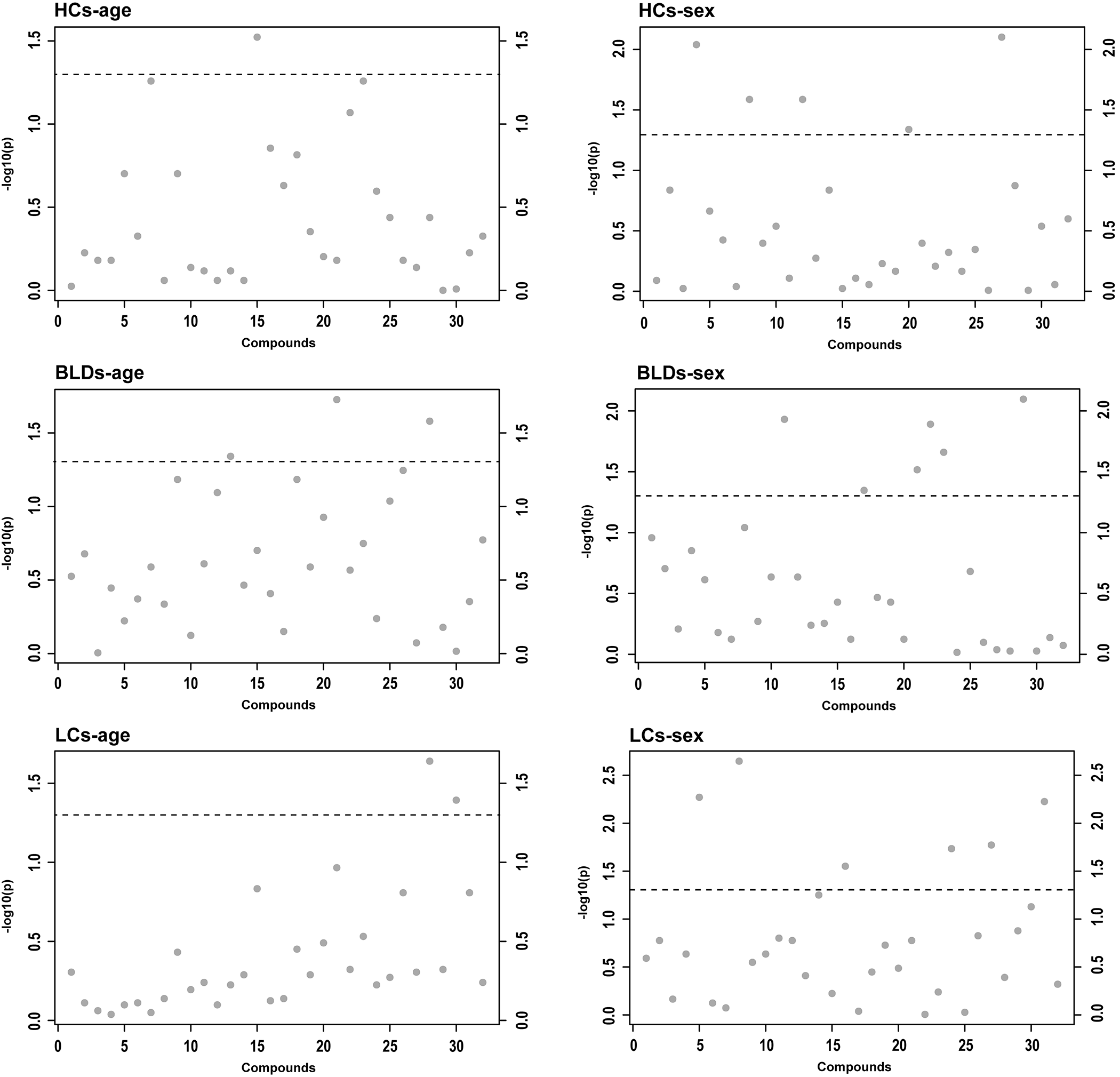


**Supplementary Figure 6.** Wilcoxon rank-sum tests for age and gender. Each dot indicates an amine, and gray indicates no significant difference.

**Supplementary Table 8.** **The concentration of amine in the serum.**

| AAs (μM) | Pathophysiological condition (n=103) | | |
| --- | --- | --- | --- |
|  | HC (n=29) | BLD (n=37) | LC (n=37) |
|  | Mean [95% CI] | Mean [95% CI] | Mean [95% CI] |
| Glycine | 410.6 [334.5-486.8] | 248.5 [231.8-265.2] | 315.9 [287.2-344.6] |
| Alanine | 562.6 [496.1-629.2] | 293.9 [256.6-331.1] | 430.6 [375.2-486.0] |
| Serine | 154.8 [141.5-168.2] | 98.84 [85.52-112.2] | 115.2 [97.53-132.8] |
| Proline | 159.9 [129.3-190.5] | 155.1 [112.8-197.4] | 122.8 [103.8-141.8] |
| Valine | 311.1 [284.0-338.3] | 292.8 [266.2-319.4] | 290.1 [273.6-306.5] |
| Threonine | 115.5 [107.2-123.9] | 102.2 [94.69-109.7] | 99.60 [93.43-105.8] |
| Taurine | 82.93 [76.03-89.83] | 62.73 [59.73-65.73] | 69.50[64.49-74.52] |
| Leucine/Isoleucine | 282.5 [257.8-307.1] | 213.7 [187.2-240.2] | 269.0 [240.6-297.3] |
| Glutamine | 470.0 [430.3-509.8] | 398.3 [365.7-431.0] | 581.0 [532.4-629.5] |
| Methionine | 12.84 [11.91-13.78] | 7.759 [7.002-8.516] | 11.49 [10.47-12.51] |
| Phenylalanine | 110.4 [103.8-117.1] | 84.55 [77.96-91.14] | 97.80 [90.29-105.3] |
| Tyrosine | 69.66 [63.23-76.09] | 61.32 [53.63-69.02] | 58.39 [53.01-63.77] |
| Tryptophan | 73.98 [68.51-79.44] | 74.94 [66.30-83.64] | 65.23 [58.86-71.59] |
| N-α-Acetyl-L-lysine | 2.208 [2.009-2.406] | 2.328 [2.120-2.536] | 2.713[2.457-2.970] |
| Citrulline | 10.32 [9.103-11.53] | 11.26 [9.919-12.59] | 14.70 [12.87-16.53] |
| Hydroxyproline | 7.196 [5.522-8.869] | 7.951 [6.042-9.860] | 10.50 [8.798-12.20] |
| Asparagine | 47.41 [44.37-50.45] | 40.50 [37.83-43.17] | 44.03 [41.35-46.71] |
| Arginine | 71.21 [64.47-77.95] | 64.73 [59.43-70.03] | 70.79 [61.98-78.62] |
| Lysine | 139.8 [130.5-149.1] | 133.1 [123.9-142.2] | 137.5 [128.4-146.5] |
| Histidine | 100.7 [87.43-113.9] | 76.74 [68.17-85.30] | 85.04 [75.99-94.09] |
| Ornithine | 63.77 [56.63-70.92] | 64.63 [55.88-73.39] | 70.30 [55.78-70.76] |
| N2-AcetylOrnithine | 2.328 [2.124-2.531] | 2.412 [2.194-2.631] | 3.497 [3.037-3.957] |
| Aspartic acid | 50.01 [45.17-54.85] | 21.27 [17.19-24.74] | 32.63 [27.32-37.94] |
| Glutamic acid | 166.5 [154.9-178.1] | 76.92 [67.85-85.99] | 102.7 [89.39-116.1] |
| GABA | 10.74 [9.783-11.69] | 8.125 [7.522-8.728] | 9.121 [8.243-9.999] |
| alanylglutamine | 7.642 [7.283-8.000] | 7.373 [6.898-7.848] | 6.909 [6.306-7.512] |
| Cystathionine | 5.019 [4.777-5.261] | 3.972 [3.846-4.098] | 5.000 [4.711-5.288] |
| 2-Aminoheptanoic acid | 1.798 [1.683-1.913] | 1.904 [1.763-2.046] | 2.197 [2.064-2.330] |
| S-(2-carboxypropyl)-Cysteamine | 3.168 [3.082-3.255] | 2.910 [2.831-2.990] | 3.249 [3.112-3.386] |
| pyroglutamine | 1.856 [1.562-2.150] | 2.372 [2.014-2.731] | 2.842 [2.476-3.208] |
| Pipecolic acid | 20.68 [18.03-23.34] | 15.96 [12.54-19.37] | 21.37 [17.26-25.48] |
| 5-Hydroxylysine | 1.820 [1.681-1.959] | 1.049 [0.9532-1.144] | 1.533 [1.383-1.683] |

**Supplementary Table 9.** Kruskal-Wallis test of amines in serum.

|  | HCs *vs.* BLDs | HCs *vs.* LCs | BLDs *vs.* LCs |
| --- | --- | --- | --- |
|  | p | p | p |
| Glycine | **<0.0001** | **0.0077** | **0.0028** |
| Alanine | **<0.0001** | **0.0126** | **0.0005** |
| Serine | **<0.0001** | **0.0004** | 0.7014 |
| Proline | 0.2825 | 0.1887 | >0.9999 |
| Valine | 0.6338 | >0.9999 | >0.9999 |
| Threonine | **0.0232** | **0.0119** | >0.9999 |
| Taurine | **<0.0001** | **0.0057** | 0.1594 |
| Leucine/Isoleucine | **0.0001** | >0.9999 | **0.0021** |
| Glutamine | **0.0447** | **0.0121** | **<0.0001** |
| Methionine | **<0.0001** | 0.2013 | **<0.0001** |
| Phenylalanine | **<0.0001** | **0.0469** | **0.0213** |
| Tyrosine | **0.0340** | **0.0327** | >0.9999 |
| Tryptophan | >0.9999 | 0.1905 | 0.4701 |
| N-α-Acetyl-L-lysine | >0.9999 | **0.0157** | 0.0898 |
| Citrulline | >0.9999 | **0.0023** | 0.0178 |
| Hydroxyproline | >0.9999 | **0.0064** | **0.0081** |
| Asparagine | **0.0012** | 0.2804 | 0.1443 |
| Arginine | 0.2434 | >0.9999 | 0.5235 |
| Lysine | 0.9631 | >0.9999 | >0.9999 |
| Histidine | **0.0007** | 0.0744 | 0.3701 |
| Ornithine | >0.9999 | 0.9613 | 0.4024 |
| N2-AcetylOrnithine | >0.9999 | **<0.0001** | **0.0001** |
| Aspartic acid | **<0.0001** | **0.0003** | **0.0107** |
| Glutamic acid | **<0.0001** | **<0.0001** | **0.0284** |
| GABA | **0.0001** | **0.0254** | 0.3300 |
| Alanylglutamine | >0.9999 | >0.9999 | >0.9999 |
| Cystathionine | **<0.0001** | >0.9999 | **<0.0001** |
| 2-Aminoheptanoic acid | 0.6338 | **<0.0001** | **0.0026** |
| S-(2-carboxypropyl)-Cysteamine | **<0.0001** | >0.9999 | **<0.0001** |
| Pipecolic acid | **0.0429** | >0.9999 | >0.0961 |
| Pyroglutamine | 0.1165 | **0.0003** | 0.1443 |
| 5-Hydroxylysine | **<0.0001** | **0.0462** | **<0.0001** |

**Supplementary Table 10.** **Amines in the biological samples.**

|  |  | Molecular formula | Theoretical  m/z  [M-H]^-^ | Observed  m/z  [M-H]^-^ | Error (ppm) | Sample |
| --- | --- | --- | --- | --- | --- | --- |
| 1 | Glycine | C_2_H_5_NO_2_ | 162.0230 | 162.0230 | 0.00 | spleen , liver , heart , kidney , lung , brain , serum , HCC-827 , NCI-H1650 , NCI-H2228 |
| 2 | Alanine | C_3_H_7_NO_2_ | 176.0387 | 176.0387 | 0.00 | spleen , liver , heart , kidney , lung , brain , serum , HCC-827 , NCI-H1650 , NCI-H2228 |
| 3 | GABA | C_4_H_9_NO_2_ | 190.0543 | 190.0544 | 0.53 | spleen , liver , heart , kidney , lung , brain , serum , HCC-827 , NCI-H1650 , NCI-H2228 |
| 4 | Serine | C_3_H_7_NO_3_ | 192.0336 | 192.0337 | 0.52 | spleen , liver , heart , kidney , lung , brain , serum , HCC-827 , NCI-H1650 , NCI-H2228 |
| 5 | Hypotaurine | C_2_H_7_NO_2_S | 196.0108 | 196.0108 | 0.00 | spleen , liver , heart , kidney , lung , brain , serum , HCC-827 , NCI-H1650 , NCI-H2228 |
| 6 | Histamine | C_5_H_9_N_3_ | 198.0707 | 198.0707 | 0.00 | spleen , heart |
| 7 | Proline | C_5_H_9_NO_2_ | 202.0543 | 202.0544 | 0.49 | spleen , liver , heart , kidney , lung , brain , serum , HCC-827 , NCI-H1650 , NCI-H2228 |
| 8 | Valine | C_5_H_11_NO_2_ | 204.0700 | 204.0701 | 0.49 | spleen , liver , heart , kidney , lung , brain , serum , HCC-827 , NCI-H1650 , NCI-H2228 |
| 9 | Threonine | C_4_H_9_NO_3_ | 206.0493 | 206.0494 | 0.49 | spleen , liver , heart , kidney , lung , brain , serum , HCC-827 , NCI-H1650 , NCI-H2228 |
| 10 | Cysteine | C_3_H_7_NO_2_S | 208.0108 | 208.0109 | 0.48 | spleen , liver , heart , kidney , lung , brain , serum , HCC-827 , NCI-H1650 , NCI-H2228 |
| 11 | Taurine | C_2_H_7_NO_3_S | 212.0057 | 212.0056 | -0.47 | spleen , liver , heart , kidney , lung , brain , serum , HCC-827 , NCI-H1650 , NCI-H2228 |
| 12 | Pyroglutamine | C_5_H_8_N_2_O_2_ | 215.0496 | 215.0497 | 0.47 | spleen , liver , heart , kidney , lung , brain , serum , HCC-827 , NCI-H1650 , NCI-H2228 |
| 13 | 4-Oxo-L-proline | C_5_H_7_NO_3_ | 216.0336 | 216.0337 | 0.46 | spleen , liver , heart , kidney , lung , brain , serum , HCC-827 , NCI-H1650 , NCI-H2228 |
| 14 | Pipecolic acid | C_6_H_11_NO_2_ | 216.0700 | 216.0700 | 0.00 | serum , HCC-827 , NCI-H1650 , NCI-H2228 |
| 15 | 3-Amino-2-pyrrolidinecarboxylic acid | C_5_H_10_N_2_O_2_ | 217.0652 | 217.0653 | 0.46 | spleen , liver , heart , kidney , lung , brain , serum , HCC-827 , NCI-H1650 , NCI-H2228 |
| 16 | Hydroxyproline | C_5_H_9_NO_3_ | 218.0493 | 218.0493 | 0.00 | spleen , liver , heart , kidney , lung , brain , serum , HCC-827 , NCI-H1650 , NCI-H2228 |
| 17 | Creatine | C_4_H_9_N_3_O_2_ | 218.0605 | 218.0606 | 0.46 | heart , kidney , serum , HCC-827 , NCI-H1650 , NCI-H2228 |
| 18 | Leucine/Isoleucine | C_6_H_13_NO_2_ | 218.0856 | 218.0857 | 0.46 | spleen , liver , heart , kidney , lung , brain , serum , HCC-827 , NCI-H1650 , NCI-H2228 |
| 19 | Asparaginate | C_4_H_8_N_2_O_3_ | 219.0445 | 219.0446 | 0.46 | spleen , liver , heart , kidney , lung , brain , serum , HCC-827 , NCI-H1650 , NCI-H2228 |
| 20 | Aspartic acid | C_4_H_7_NO_4_ | 220.0285 | 220.0286 | 0.45 | spleen , liver , heart , kidney , lung , brain , serum , HCC-827 , NCI-H1650 , NCI-H2228 |
| 21 | O-Phosphoethanolamine | C_2_H_8_NO_4_P | 228.0101 | 228.0102 | 0.44 | spleen , liver , heart , kidney , lung , brain |
| 22 | Histidinol | C_6_H_11_N_3_O | 228.0812 | 228.0813 | 0.44 | spleen , liver , heart , kidney , lung , brain , serum , HCC-827 , NCI-H1650 , NCI-H2228 |
| 23 | 2-Aminoheptanoic acid | C_7_H_15_NO_2_ | 232.1013 | 232.1012 | -0.43 | spleen , liver , kidney , lung , serum , HCC-827 , NCI-H1650 , NCI-H2228 |
| 24 | Glutamine | C_5_H_10_N_2_O_3_ | 233.0602 | 233.0602 | 0.00 | spleen , liver , heart , kidney , lung , brain , serum , HCC-827 , NCI-H1650 , NCI-H2228 |
| 25 | Glutamic acid | C_5_H_9_NO_4_ | 234.0442 | 234.0442 | 0.00 | spleen , liver , heart , kidney , lung , brain , serum , HCC-827 , NCI-H1650 , NCI-H2228 |
| 26 | 2-Amino-4-hydroxy-3-methylpentanoic acid | C_6_H_13_NO_3_ | 234.0806 | 234.0806 | 0.00 | spleen , liver , heart , kidney , lung , brain , serum , HCC-827 , NCI-H1650 , NCI-H2228 |
| 27 | Methionine | C_5_H_11_O_2_NS | 236.0421 | 236.0421 | 0.00 | spleen , liver , heart , kidney , lung , brain , serum , HCC-827 , NCI-H1650 , NCI-H2228 |
| 28 | Dopamine | C_8_H_11_NO_2_ | 240.0700 | 240.0701 | 0.42 | brain |
| 29 | Histidine | C_6_H_9_N_3_O_2_ | 242.0605 | 242.0606 | 0.41 | spleen , liver , heart , kidney , lung , brain , serum , HCC-827 , NCI-H1650 , NCI-H2228 |
| 30 | L-2-Amino-4-methylenepentanedioic acid | C_6_H_9_NO_4_ | 246.0442 | 246.0442 | 0.00 | spleen , liver , heart , kidney , lung , brain , HCC-827 , NCI-H1650 , NCI-H2228 |
| 31 | Methyl 5-(hydroxymethyl)pyrrolidine-3-carboxylate | C_7_H_13_NO_3_ | 246.0806 | 246.0806 | 0.00 | spleen , liver , heart , kidney , lung , brain , serum , HCC-827 , NCI-H1650 , NCI-H2228 |
| 32 | DL-2-Aminooctanoic acid | C_8_H_17_NO_2_ | 246.1169 | 246.1170 | 0.41 | kidney , serum , HCC-827 , NCI-H1650 , NCI-H2228 |
| 33 | N-formimidoyl-L-aspartic acid | C_5_H_8_N_2_O_4_ | 247.0394 | 247.0394 | 0.00 | spleen , liver , heart , kidney , lung , brain , NCI-H1650 |
| 34 | Aminoadipic acid | C_6_H_11_NO_4_ | 248.0598 | 248.0598 | 0.00 | spleen , liver , heart , kidney , lung , brain , serum , HCC-827 , NCI-H1650 , NCI-H2228 |
| 35 | 5-Hydroxylysine | C_6_H_14_N_2_O_3_ | 249.0915 | 249.0914 | -0.40 | spleen , liver , heart , kidney , lung , brain , serum , HCC-827 , NCI-H1650 , NCI-H2228 |
| 36 | S-(2-carboxypropyl)-Cysteamine | C_6_H_13_NO_2_S | 250.0577 | 250.0576 | -0.40 | spleen , liver , heart , kidney , lung , brain , serum , NCI-H827 , NCI-H1650 |
| 37 | 2-Amino-4-ethoxy-3-hydroxybutanoic acid | C_6_H_13_NO_4_ | 250.0755 | 250.0754 | -0.40 | liver , kidney , serum , NCI-H827 , NCI-H1650 |
| 38 | Methionine sulfoxide | C_5_H_11_NO_3_S | 252.0370 | 252.0370 | 0.00 | spleen , liver , heart , kidney , lung , brain , serum , HCC-827 , NCI-H1650 , NCI-H2228 |
| 39 | Phenylalanine | C_9_H_11_NO_2_ | 252.0700 | 252.0701 | 0.40 | spleen , liver , heart , kidney , lung , brain , serum , HCC-827 , NCI-H1650 , NCI-H2228 |
| 40 | Norepinephrine | C_8_H_11_NO_3_ | 256.0649 | 256.0650 | 0.39 | kidney , brain , serum , NCI-H827 |
| 41 | Methylhistidine | C_7_H_11_N_3_O_2_ | 256.0761 | 256.0762 | 0.39 | spleen , liver , heart , kidney , lung , brain , serum , HCC-827 , NCI-H1650 , NCI-H2228 |
| 42 | Prolylglycine | C_7_H_12_N_2_O_3_ | 259.0758 | 259.0758 | 0.00 | spleen , liver , heart , kidney , lung , brain , serum , HCC-827 , NCI-H1650 , NCI-H2228 |
| 43 | 2-Oxoarginine | C_6_H_11_N_3_O_3_ | 260.0711 | 260.0710 | -0.38 | spleen , liver , heart , kidney , lung , brain |
| 44 | Formiminoglutamic acid | C_6_H_10_N_2_O_4_ | 261.0551 | 261.0552 | 0.38 | spleen、liver、heart、kidney、lung、brain , HCC-827 , NCI-H1650 , NCI-H2228 |
| 45 | N2-Acetylornithine | C_7_H_14_N_2_O_3_ | 261.0915 | 261.0915 | 0.00 | spleen , liver , heart , kidney , lung , brain , serum , HCC-827 , NCI-H1650 , NCI-H2228 |
| 46 | Arginine | C_6_H_14_N_4_O_2_ | 261.1027 | 261.1028 | 0.38 | spleen , liver , heart , kidney , lung , brain , serum , HCC-827 , NCI-H1650 , NCI-H2228 |
| 47 | Guanidinosuccinic acid | C_5_H_9_N_3_O_4_ | 262.0503 | 262.0504 | 0.38 | spleen , kidney , lung , serum , HCC-827 , NCI-H1650 , NCI-H2228 |
| 48 | 2-Aminoheptanedioic acid | C_7_H_13_NO_4_ | 262.0755 | 262.0754 | -0.38 | spleen , heart , kidney , lung , brain , serum , HCC-827 , NCI-H1650 , NCI-H2228 |
| 49 | Citrulline | C_6_H_13_N_3_O_3_ | 262.0867 | 262.0868 | 0.38 | spleen , liver , heart , kidney , lung , brain , serum , HCC-827 , NCI-H1650 , NCI-H2228 |
| 50 | Alanylserine/Glycyl-Threonine | C_6_H_12_N_2_O_4_ | 263.0707 | 263.0708 | 0.38 | spleen , liver , heart , kidney , lung , brain , serum , HCC-827 , NCI-H1650 , NCI-H2228 |
| 51 | Serotonin | C_10_H_12_N_2_O | 263.0860 | 263.0859 | -0.38 | spleen , HCC-827 , NCI-H1650 , NCI-H2228 |
| 52 | S-(1-Propenyl)-cysteine sulfoxide | C_6_H_11_NO_3_S | 264.0370 | 264.0370 | 0.00 | spleen , liver , heart , kidney , lung , brain , serum , HCC-827 , NCI-H1650 , NCI-H2228 |
| 53 | O-Ureidohomoserine | C_5_H_11_N_3_O_4_ | 264.0660 | 264.0659 | -0.38 | spleen , liver , heart , kidney , lung , brain , serum , HCC-827 , NCI-H1650 , NCI-H2228 |
| 54 | Glucosamine | C_6_H_13_NO_5_ | 266.0704 | 266.0704 | 0.00 | spleen , liver , heart , kidney , lung , brain , serum , HCC-827 , NCI-H1650 , NCI-H2228 |
| 55 | Methionine sulfoximine | C_5_H_12_N_2_O_3_S | 267.0479 | 267.0479 | 0.00 | spleen , liver , heart , kidney , lung , brain , serum , HCC-827 , NCI-H1650 , NCI-H2228 |
| 56 | DL-Methionine sulfone | C_5_H_11_NO_4_S | 268.0319 | 268.0319 | 0.00 | spleen , liver , heart , kidney , lung , brain , HCC-827 , NCI-H1650 , NCI-H2228 |
| 57 | Tyrosine | C_9_H_11_NO_3_ | 268.0649 | 268.0649 | 0.00 | spleen , liver , heart , kidney , lung , brain , serum , HCC-827 , NCI-H1650 , NCI-H2228 |
| 58 | 2,6-Diamino-4-hydroxy-5-N-methylformamidopyrimidine | C_6_H_9_N_5_O_2_ | 270.0666 | 270.0668 | 0.74 | liver , heart , kidney , serum , HCC-827 , NCI-H1650 , NCI-H2228 |
| 59 | O-Phospho-L-serine | C_3_H_8_NO_6_P | 271.9999 | 272.0000 | 0.37 | HCC-827 , NCI-H1650 , NCI-H2228 |
| 60 | 2-Hepteneoylglycine | C_9_H_15_NO_3_ | 272.0962 | 272.0962 | 0.00 | serum , HCC-827 , NCI-H1650 , NCI-H2228 |
| 61 | Prolyl-Alanine | C_8_H_14_N_2_O_3_ | 273.0915 | 273.0914 | -0.37 | spleen , liver , kidney , lung , brain , serum , HCC-827 , NCI-H1650 , NCI-H2228 |
| 62 | N-alpha-Acetyl-L-lysine | C_8_H_16_N_2_O_3_ | 275.1071 | 275.1071 | 0.00 | spleen , liver , heart , kidney , lung , brain , serum , HCC-827 , NCI-H1650 , NCI-H2228 |
| 63 | Homo-L-arginine | C_7_H_16_N_4_O_2_ | 275.1183 | 275.1185 | 0.73 | brain , serum , HCC-827 , NCI-H1650 |
| 64 | Homocitrulline | C_7_H_15_N_3_O_3_ | 276.1024 | 276.1024 | 0.00 | spleen , liver , heart , kidney , lung , brain , serum , HCC-827 , NCI-H1650 , NCI-H2228 |
| 65 | N-carbamoylglutamic Acid/Aspartyl-Glycine | C_6_H_10_N_2_O_5_ | 277.0500 | 277.0499 | -0.36 | spleen , liver , heart , kidney , lung , brain , serum , HCC-827 , NCI-H1650 , NCI-H2228 |
| 66 | meso-2,6-Diaminoheptanedioate | C_7_H_14_N_2_O_4_ | 277.0864 | 277.0864 | 0.00 | spleen , liver , heart , kidney , lung , brain , serum , HCC-827 , NCI-H1650 , NCI-H2228 |
| 67 | Serylproline/Prolyl-Serine | C_8_H_14_N_2_O_4_ | 289.0864 | 289.0864 | 0.00 | spleen , liver , kidney , brain , serum , HCC-827 , NCI-H1650 , NCI-H2228 |
| 68 | N-Acetylisoputreanine | C_9_H_18_N_2_O_3_ | 289.1228 | 289.1228 | 0.00 | spleen , liver , heart , kidney , lung , brain , serum , HCC-827 , NCI-H1650 , NCI-H2228 |
| 69 | Alanylasparagine/Glutaminylglycine | C_7_H_13_N_3_O_4_ | 290.0816 | 290.0816 | 0.00 | spleen , liver , heart , kidney , lung , brain , serum , HCC-827 , NCI-H1650 , NCI-H2228 |
| 70 | Alanyl-Aspartic acid/Aspartyl-Alanine | C_7_H_12_N_2_O_5_ | 291.0656 | 291.0656 | 0.00 | spleen , liver , heart , kidney , lung , brain , serum , HCC-827 , NCI-H1650 , NCI-H2228 |
| 71 | Tryptophan | C_11_H_12_N_2_O_2_ | 291.0809 | 291.0808 | -0.34 | spleen , liver , heart , kidney , lung , brain , serum , HCC-827 , NCI-H1650 |
| 72 | Glycyl-Methionine | C_7_H_14_N_2_O_3_S | 293.0635 | 293.0636 | 0.34 | spleen , liver , heart , kidney , lung , brain , serum , HCC-827 , NCI-H1650 , NCI-H2228 |
| 73 | Serylthreonine/Threonylserine | C_7_H_14_N_2_O_5_ | 293.0813 | 293.0812 | -0.34 | spleen , liver , kidney , lung , brain , serum , HCC-827 , NCI-H1650 , NCI-H2228 |
| 74 | (2R,2'S)-Isobuteine | C_7_H_13_NO_4_S | 294.0475 | 294.0476 | 0.34 | spleen , liver , heart , kidney , lung , brain , serum , HCC-827 , NCI-H1650 , NCI-H2228 |
| 75 | 6-Carboxy-5,6,7,8-tetrahydropterin | C_7_H_9_N_5_O_3_ | 298.0615 | 298.0614 | -0.34 | spleen , liver , heart , kidney , lung , brain , serum , HCC-827 , NCI-H1650 , NCI-H2228 |
| 76 | Glycerylphosphorylethanolamine | C_5_H_14_NO_6_P | 302.0469 | 302.0468 | -0.33 | spleen , liver , heart , kidney , lung , brain , serum , HCC-827 , NCI-H1650 , NCI-H2228 |
| 77 | Alanylglutamine | C_8_H_15_N_3_O_4_ | 304.0973 | 304.0972 | -0.33 | spleen , liver , heart , kidney , lung , brain , serum , HCC-827 , NCI-H1650 , NCI-H2228 |
| 78 | Alanylglutamic acid/Glutamylalanine | C_8_H_14_N_2_O_5_ | 305.0813 | 305.0812 | -0.33 | spleen , liver , heart , kidney , lung , brain , serum , HCC-827 , NCI-H1650 , NCI-H2228 |
| 79 | Threoninyl-Valine/Threonylvaline | C_9_H_18_N_2_O_4_ | 305.1177 | 305.1176 | -0.33 | spleen , liver , heart , kidney , lung , brain , serum , HCC-827 , NCI-H1650 , NCI-H2228 |
| 80 | Aspartyl-Serine | C_7_H_12_N_2_O_6_ | 307.0605 | 307.0604 | -0.33 | spleen , liver , heart , kidney , lung , brain |
| 81 | Ornithine | C_5_H_12_N_2_O_2_ | 307.0792 | 307.0791 | -0.33 | spleen , liver , heart , kidney , lung , brain , serum , HCC-827 , NCI-H1650 , NCI-H2228 |
| 82 | 2'-Deoxysepiapterin | C_9_H_11_N_5_O_2_ | 308.0826 | 308.0826 | 0.00 | liver , kidiney , lung , serum |
| 83 | Cystathionine | C_7_H_14_N_2_O_4_S | 309.0584 | 309.0584 | 0.00 | spleen , liver , heart , kidney , lung , brain , serum , HCC-827 , NCI-H1650 , NCI-H2228 |
| 84 | (2S,3'S)-alpha-Amino-2-carboxy-5-oxo-1-pyrrolidinebutanoic acid | C_9_H_14_N_2_O_5_ | 317.0813 | 317.0816 | 0.95 | serum , HCC-827 , NCI-H1650 , NCI-H2228 |
| 85 | N2-Succinyl-L-ornithine | C_9_H_16_N_2_O_5_ | 319.0969 | 319.0970 | 0.31 | spleen , liver , heart , kidney , lung , brain , serum , HCC-827 , NCI-H1650 , NCI-H2228 |
| 86 | Asparaginyl-Threonine/Threonylasparagine | C_8_H_15_N_3_O_5_ | 320.0922 | 320.0921 | -0.31 | spleen , liver , heart , kidney , lung , brain , serum , HCC-827 , NCI-H1650 , NCI-H2228 |
| 87 | Hydroxyprolyl-Cysteine | C_8_H_14_N_2_O_4_S | 321.0584 | 321.0584 | 0.00 | spleen , liver , heart , kidney , lung , brain , serum , HCC-827 , NCI-H1650 , NCI-H2228 |
| 88 | Lysine | C_6_H_14_N_2_O_2_ | 321.0948 | 321.0947 | -0.31 | spleen , liver , heart , kidney , lung , brain , serum , HCC-827 , NCI-H1650 , NCI-H2228 |
| 89 | Methionyl-Serine/Serylmethionine | C_8_H_16_N_2_O_4_S | 323.0741 | 323.0740 | -0.31 | spleen , liver , heart , kidney , lung , brain , serum , HCC-827 , NCI-H1650 , NCI-H2228 |
| 90 | Biopterin | C_9_H_11_N_5_O_3_ | 324.0772 | 324.0771 | -0.31 | spleen , liver , heart , kidney , lung , brain , serum , HCC-827 , NCI-H1650 , NCI-H2228 |
| 91 | Cystathionine sulfoxide | C_7_H_14_N_2_O_5_S | 325.0534 | 325.0532 | -0.62 | spleen , liver , heart , kidney , lung , brain , serum , HCC-827 , NCI-H1650 , NCI-H2228 |
| 92 | Dihydrobiopterin | C_9_H_13_N_5_O_3_ | 326.0928 | 326.0929 | 0.31 | spleen , liver , heart , kidney , lung , brain , serum , HCC-827 , NCI-H1650 , NCI-H2228 |
| 93 | Anserine | C_10_H_16_N_4_O_3_ | 327.1132 | 327.1135 | 0.92 | spleen , liver , heart , kidney , lung , brain |
| 94 | Threonylglutamine/Threoninyl-Gamma-glutamate | C_9_H_17_N_3_O_5_ | 334.1078 | 334.1078 | 0.00 | spleen , liver , heart , kidney , lung , brain , serum , HCC-827 , NCI-H1650 , NCI-H2228 |
| 95 | N(6)-Methyllysine | C_7_H_16_N_2_O_2_ | 335.1105 | 335.1103 | -0.60 | spleen , liver , heart , kidney , lung , brain , serum , HCC-827 , NCI-H1650 , NCI-H2228 |
| 96 | Gamma-Glutamylcysteine | C_8_H_14_N_2_O_5_S | 337.0534 | 337.0532 | -0.59 | spleen , liver , heart , kidney , lung , brain , serum , HCC-827 , NCI-H1650 , NCI-H2228 |
| 97 | Aspartyl-Lysine | C_10_H_19_N_3_O_5_ | 348.1235 | 348.1233 | -0.57 | spleen , liver , heart , kidney , lung , brain , serum , HCC-827 , NCI-H1650 , NCI-H2228 |
| 98 | Asparaginyl-Methionine/Methionyl-Asparagine | C_9_H_17_N_3_O_4_S | 350.0850 | 350.0849 | -0.29 | spleen , liver , heart , kidney , lung , brain , serum , HCC-827 , NCI-H1650 , NCI-H2228 |
| 99 | Homocystine | C_8_H_16_N_2_O_4_S_2_ | 355.0462 | 355.0463 | 0.28 | spleen , liver , lung , brain , serum , HCC-827 , NCI-H1650 , NCI-H2228 |
| 100 | N2-Succinyl-L-arginine | C_10_H_18_N_4_O_5_ | 361.1187 | 361.1186 | -0.28 | liver , HCC-827 |
| 101 | Argininosuccinic acid | C_10_H_18_N_4_O_6_ | 377.1136 | 377.1135 | -0.27 | spleen , liver , heart , kidney , lung , brain , serum , HCC-827 , NCI-H1650 , NCI-H2228 |
| 102 | Spermine | C_10_H_26_N_4_ | 377.2050 | 377.2050 | 0.00 | HCC-827 |
| 103 | Glutathione | C_10_H_17_N_3_O_6_S | 394.0748 | 394.0745 | -0.76 | spleen , liver , heart , kidney , lung , brain , serum , HCC-827 , NCI-H1650 , NCI-H2228 |
